# Triangulating associations between fruit intake and lung cancer risk: evidence from GBD estimates, Mendelian randomization, and real-world validation

**DOI:** 10.1093/oncolo/oyag069

**Published:** 2026-02-27

**Authors:** Mengqing Liu, Qilin Hu, Xinyue Zhang, Zhaoqi Wu, Shiwei Wang, Yile Hu, Zhe Xu, Ji Luo, Lei Sun

**Affiliations:** Department of Medical Oncology, The First Hospital of China Medical University, Shenyang 110001, China; Department of Nutrition and Food Hygiene, School of Public Health, China Medical University, Shenyang 110001, China; Department of Medical Oncology, The First Hospital of China Medical University, Shenyang 110001, China; Department of Medical Oncology, The First Hospital of China Medical University, Shenyang 110001, China; Department of Anesthesiology, The First Hospital of China Medical University, Shenyang 110001, China; Department of Pathology, The First Hospital of China Medical University, Shenyang 110001, China; Department of Thoracic Surgery, The First Hospital of China Medical University, Shenyang 110001, China; Department of Thoracic Surgery, The First Hospital of China Medical University, Shenyang 110001, China; Department of Thoracic Surgery, The First Hospital of China Medical University, Shenyang 110001, China; Department of Thoracic Surgery, The First Hospital of China Medical University, Shenyang 110001, China

**Keywords:** epidemiology, global burden, GBD 2021, prediction, TBL, mendelian randomization

## Abstract

**Background:**

Lung cancer is the leading cause of cancer-related deaths globally, with dietary factors such as low fruit intake potentially contributing to regional disparities. However, establishing causality remains challenging. This study utilized a triangulated approach combining Global Burden of Disease (GBD) data, Mendelian randomization (MR), and a hospital-based cohort to investigate the association between fruit intake and lung cancer risk.

**Methods:**

GBD 2021 data (1990-2021) were analyzed by sex, age, and socio-demographic index (SDI) using age–period–cohort models, decomposition, and ARIMA forecasting. Two-sample MR using UK Biobank instruments for fresh and dried fruit was applied to European GWAS of lung cancer and subtypes, with IVW as the primary estimator with multiple sensitivity analyses. Findings were validated in a Chinese hospital cohort (n = 641) using a food frequency questionnaire, multivariable logistic regression, and a composite Non-Diet Risk Index (NDRI).

**Findings:**

In 2021, approximately 66 000 deaths and 1.44 million DALYs were attributable to low-fruit diets. Although absolute numbers rose since 1990, age-standardized rates declined. The burden was highest in medium-SDI regions, with notable geographic and sex disparities. ARIMA projections indicate a continued decline in global rates by 2050, particularly among males. MR supported a protective effect of genetically predicted fruit intake, though sensitivity analyses showed some inconsistency. In the Chinese cohort, higher fruit intake remained significantly protective after adjusting for key confounders, including NDRI.

**Conclusion:**

This multi-method study strengthens evidence that insufficient fruit intake may increase lung cancer risk, with consistent findings across population-level, genetic, and clinical data.

Implications for Practice This study provides a theoretical foundation for the proposition that insufficient fruit intake may increase the risk of lung cancer, offering reliable theoretical support for future clinical treatment and disease prevention in lung cancer patients.

## Introduction

Lung cancer remains the leading cause of cancer-related deaths worldwide. In 2022, there were approximately 2.48 million new cases and 1.8 million deaths globally, accounting for a significant proportion of the total global cancer burden.^1^ Notably, driven by population growth, aging, and the continued rise in risk factors, the global number of new cancer cases is projected to exceed 35 million by 2050, representing a 77% increase compared to 2022.^2^ Despite advances in screening and treatment, lung cancer mortality continues to rise, reflecting demographic pressures and persistent exposure to established risk factors. Smoking remains the dominant contributor; however, accumulating evidence indicates that diet also plays an important role in lung carcinogenesis. Diets high in processed meats and saturated fats and low in fiber have been consistently linked to elevated risk, whereas fruits and vegetables, rich in antioxidants, vitamins, and phytochemicals, are considered to provide protective benefit.^3–5^ Fruits provide bioactive compounds that mitigate oxidative stress and DNA damage in lung tissues,^6^^,^^7^ offering a plausible biological basis for protection.

Nevertheless, epidemiological findings remain inconsistent.^8^^,^^9^ Many observational studies are limited by residual confounding, self-reported diet data, and inadequate adjustment for major behavioral factors such as smoking and alcohol intake. Moreover, heterogeneity in fruit type, regional dietary patterns, and population structure complicates interpretation. These uncertainties highlight the need for a comprehensive evaluation that integrates causal inference, population-level burden estimation, and individual-level validation.

To address these gaps, we adopted a triangulated study design combining three complementary analytical perspectives. First, using data from the Global Burden of Disease (GBD) 2021 study, we quantified the long-term global burden of lung cancer attributable to low fruit intake across regions and socio-demographic strata. Second, Mendelian randomization (MR) analyses were applied to assess whether genetic predisposition to lower fruit intake is associated with increased lung cancer risk, providing a quasi-causal perspective less susceptible to confounding. Finally, a hospital-based dietary survey was conducted in a Chinese population to validate the findings at the individual level and examine fruit-type-specific associations using a standardized food-frequency questionnaire.

By integrating global epidemiological modelling, genetic causal inference, and real-world validation, this study aims to provide a comprehensive understanding of how fruit consumption relates to lung cancer risk. The triangulation of evidence allows cross-verification of results from conceptually distinct but complementary methodologies, enhancing robustness and translational relevance.

## Methods

Detailed descriptions of the Materials and Methods have been moved to the Supplementary Material to enhance readability, while maintaining full transparency and reproducibility.

## Results

### Global TBL burden associated with low-fruit diets and its temporal trends

Initially, we analyzed the global tracheal, bronchial, and lung (TBL) cancer burden attributable to low fruit intake using the GBD 2021 dataset. Between 1990 and 2021, the global population expanded from 5.3 to 7.9 billion, and the contribution of dietary risks to cancer burden became more apparent (Table S1—see online supplementary material).

In 2021, an estimated 66 000 deaths (95% UI: 34 000-97 000) and 1.44 million DALYs (95% UI: 0.72-2.12 million) were attributable to low-fruit diets. Although the absolute numbers increased, age-standardized mortality (ASMR) and DALY rates (ASDR) declined with estimated annual percentage changes (EAPC) of −1.79 (95% UI: −2.69 to −0.87) and −2.11 (95% UI: −3.00 to −1.22), respectively.

The highest absolute burden occurred in medium-SDI regions, whereas the highest rates were seen in low-middle SDI countries. Males exhibited higher age-standardized rates than females. Notably, despite the decline in per-capita burden, population growth and aging largely accounted for the increasing absolute deaths.

### Regional variations and trends in TBL burden associated with low-fruit diets

**Figure 1:**
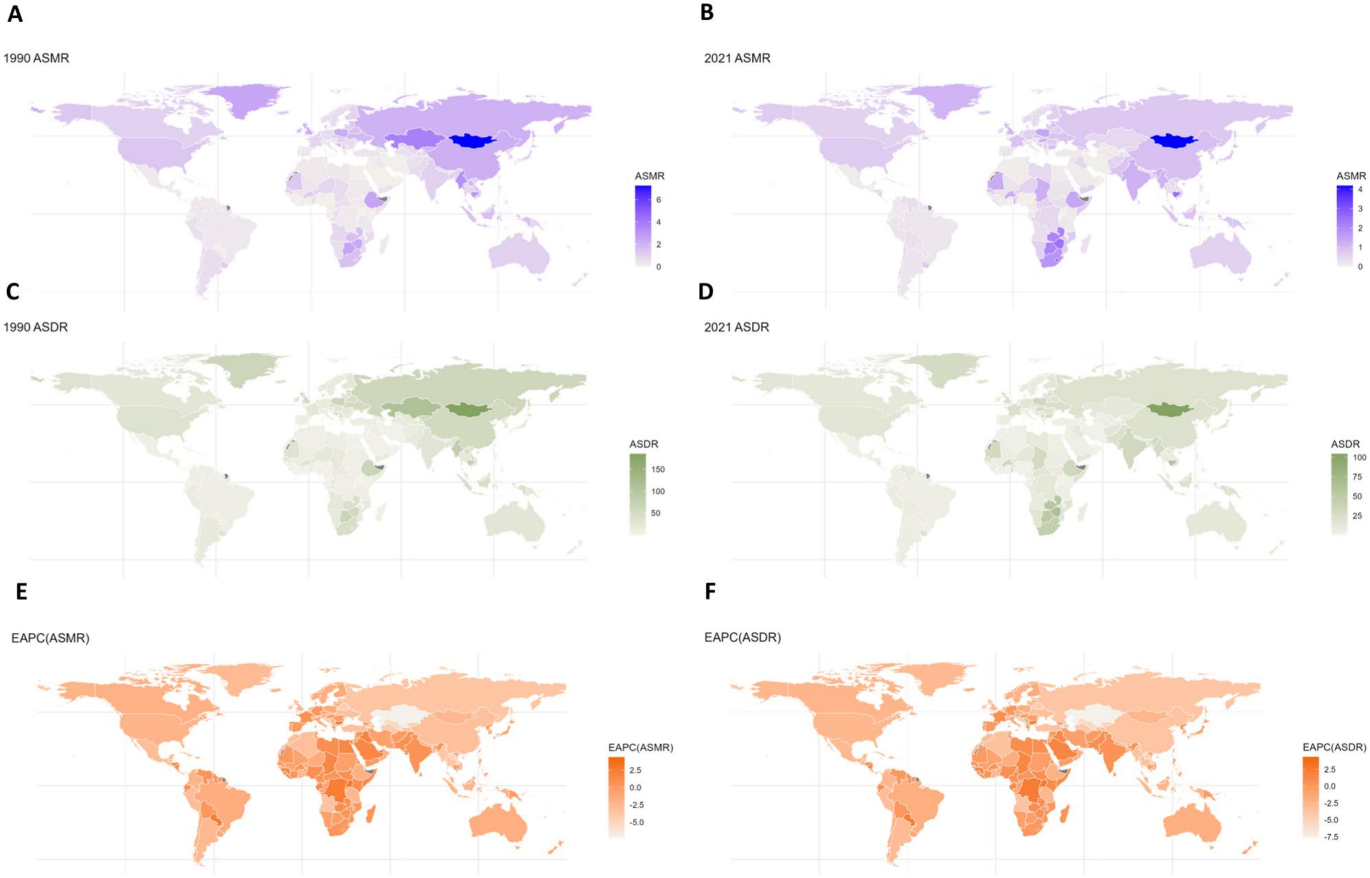
Regional variations and trends in TBL burden attributable to low-fruit diets. (A) TBL ASMR attributable to low-fruit diet in 1990. (B) TBL ASMR attributable to low-fruit diet in 2021. (C) TBL ASDR attributable to low-fruit diet in 1990. (D) TBL ASDR attributable to low-fruit diet in 2021. (E) TBL EAPC attributable to low-fruit diet from 1990-2021 (ASMR). (F) TBL EAPC (ASDR) attributable to low-fruit diets, 1990-2021.

From 1990 to 2021, substantial regional and SDI-related disparities in TBL burden attributable to low fruit intake were observed. Globally, ASMR and ASDR declined compared with 1990 (Figure 1A-D). However, despite decreasing age-standardized rates, absolute deaths increased across all regions due to population growth and ageing. Similarly, although mortality rates declined, total DALYs rose, indicating continued growth in healthy life years lost.

At the SDI level, medium-SDI regions bore the greatest absolute burden in 2021, with 0.02 million deaths and 0.53 million DALYs, accounting for over 30% of the global total, whereas low-SDI regions had the lowest burden (2 800 deaths; 0.008 million DALYs) (Table S1—see online supplementary material). The highest ASMR and ASDR were observed in low–middle SDI regions, while the lowest rates occurred in low SDI regions.

Regional analyses revealed marked heterogeneity. South Asia (16 739 deaths; 469 725 DALYs) and East Asia (19 553 deaths; 455 284 DALYs) carried the heaviest burden, whereas Oceania (57 deaths; 1 643 DALYs) and Andean Latin America (128 deaths; 3 053 DALYs) had relatively low burdens (Table S2—see online supplementary material). Although most regions experienced declining ASMR and ASDR, trends varied considerably. Central Asia showed the steepest decline in ASMR (EAPC = −4.70, 95% CI: −5.66 to −3.73), whereas sub-Saharan Africa (EAPC = 0.68, 95% CI: –0.14 to 1.51) and South Asia (EAPC = 0.47, 95% CI: −0.26 to 1.20) exhibited increasing trends (Figure 1E, F; Table S2—see online supplementary material).

At the national level, Lebanon had the largest increases in ASMR and ASDR (EAPC = 4.39, 95% CI: 3.4 to 5.4; EAPC = 4.36, 95% CI: 3.44 to 5.3), whereas Kazakhstan showed the steepest declines (EAPC = −7.3, 95% CI: −8.8 to −5.78; EAPC = −7.68, 95% CI: −9.18 to −6.15) (Figure 1E, F; Figure S2—see online supplementary material for a color version of this figure).

Across 204 countries, ASMR was not significantly associated with SDI (*P* = .8) (Figure S3A—see online supplementary material for a color version of this figure). However, at the regional level (21 GBD regions), a significant negative correlation was observed, with higher SDI generally associated with lower ASMR, particularly in East Asia, high-income Asia-Pacific, and sub-Saharan Africa (Figure S3B—see online supplementary material for a color version of this figure), suggesting a potential protective role of socioeconomic development.

### Age and sex variations in TBL burden associated with low-fruit diets

In 2021, males had higher TBL deaths and DALYs than females across all age groups globally. Among females, deaths peaked at ages 65-69, whereas among males the peak occurred at 70-74; DALYs for both sexes peaked at 65-69 (Figures S4A and S5A—see online supplementary material for a color version of these figures). Age-specific mortality increased with advancing age, consistently higher in males. Although male mortality declined after 90-94 years, it remained above female levels, while female mortality rose steadily with age (Figure S4B—see online supplementary material for a color version of this figure). DALY rates attributable to low fruit intake followed similar patterns, with persistently higher rates in males (Figure S5B—see online supplementary material for a color version of this figure).

Joinpoint regression demonstrated increasing absolute deaths from 1990 to 2021 (combined AAPC = 0.8, 95% CI: 0.8-0.8; females AAPC = 1.4, 95% CI: 1.4-1.5; males AAPC = 0.5, 95% CI: 0.5-0.6; all *P* < .001). In contrast, age-standardized mortality rates declined (combined AAPC = −1.7, 95% CI: −1.7 to −1.7; females AAPC = −1.1, 95% CI: −1.1 to −1.0; males AAPC = −2.0, 95% CI: −2.0 to −1.9; all *P* < .001), with similar trends observed for DALYs (Figures S4C, D and S5C, D—see online supplementary material for a color version of these figures).

**Figure 2:**
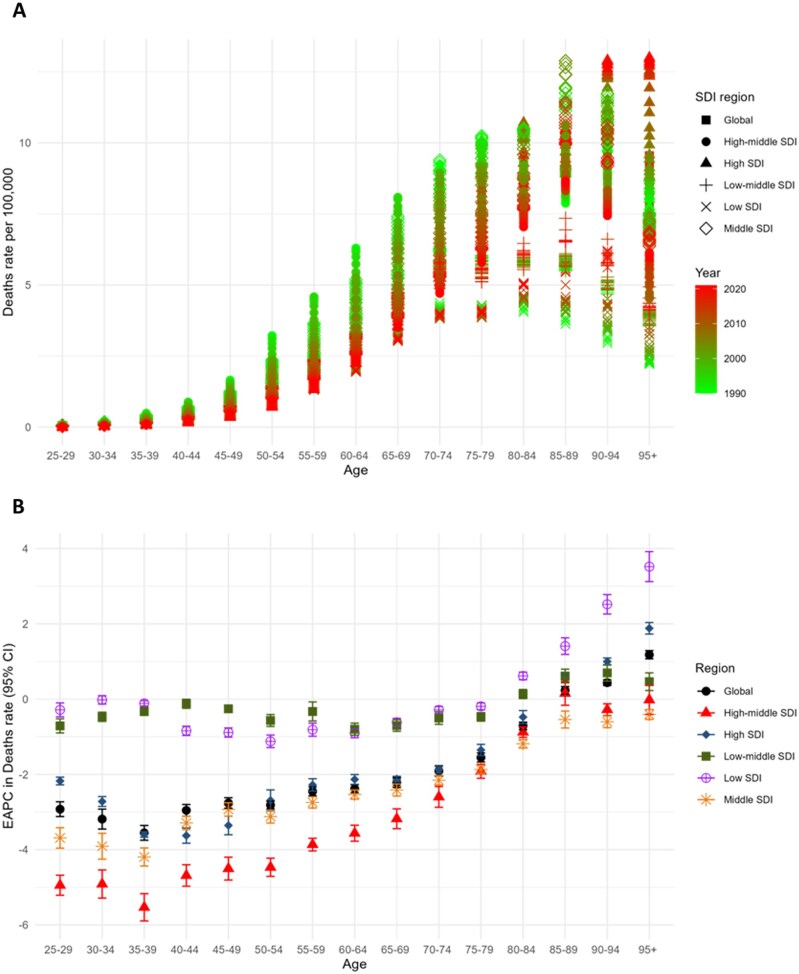
Low-fruit diet-associated TBL burden of mortality in different age groups and in different SDI regions. (A) Trends in mortality rates across SDI regions in different age groups, 1990-2021. (B) Trends in mortality EAPC across SDI regions in different age groups, 1990-2021.

From 1990 to 2021, mortality increased with age, particularly among individuals aged ≥60 years (Figure 2A). Middle-aged adults (35-49 years) showed moderate mortality with stable declines, while older populations exhibited higher but recently decreasing rates, possibly reflecting improvements in medical care. Regionally, declines were slower in low-SDI areas and faster in high-SDI regions; DALY patterns were comparable (Figure S6A—see online supplementary material for a color version of these figures).

EAPC analysis indicated declining mortality (EAPC < 0) across all SDI regions below age 80 (Figure 2B). However, in medium-low and low-SDI regions, declines were slower than the global average, and mortality increased after age 80 (EAPC > 0). In contrast, medium-SDI regions maintained EAPC < 0 across all age groups. DALY trends mirrored mortality patterns (Figure S6B—see online supplementary material for a color version of these figures).

### Age–period–cohort effects on the burden of TBL associated with low-fruit diets

**Figure 3:**
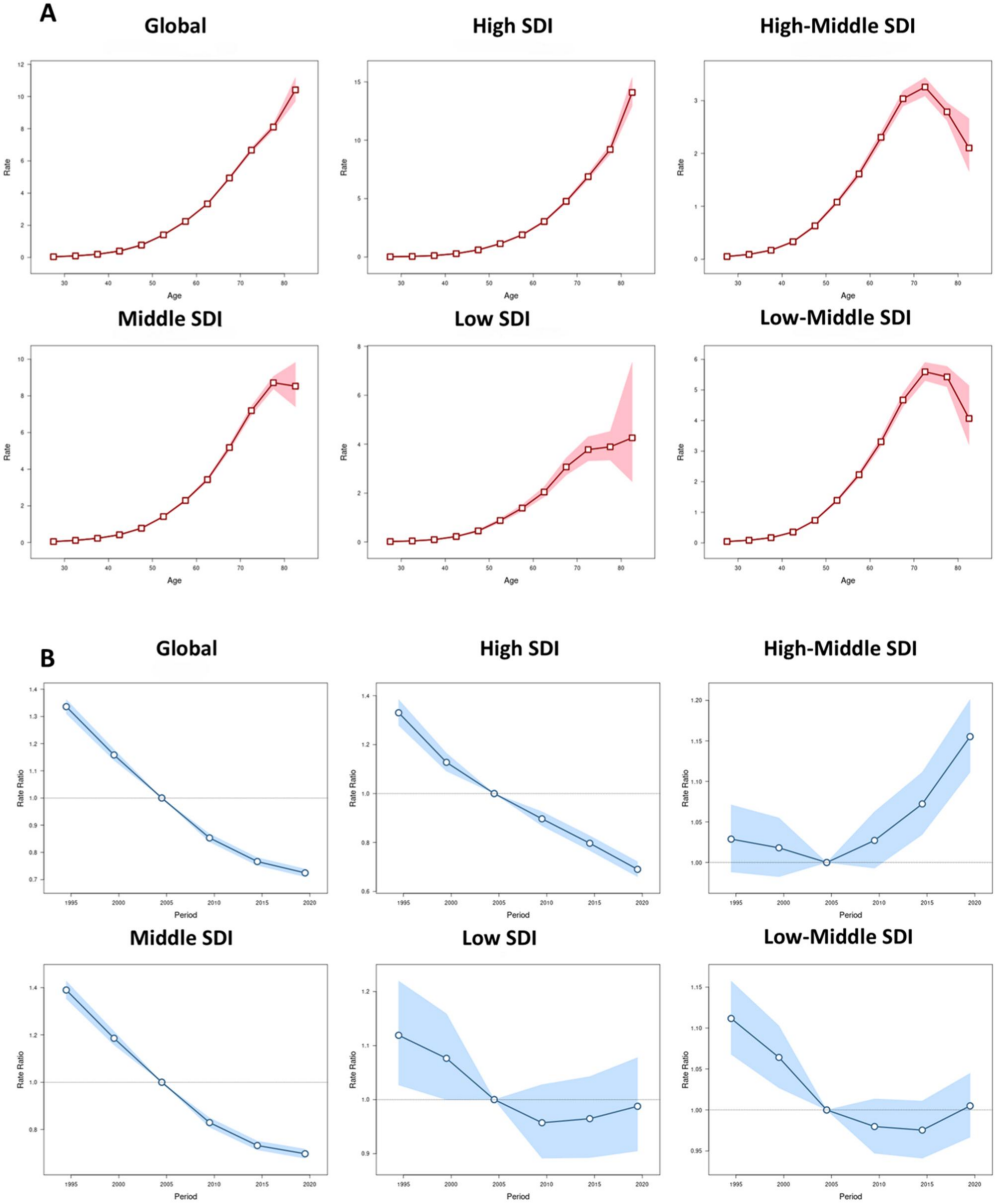
Age–period–cohort effects on TBL burden associated with low-fruit diets. (A) Trends in cross-sectional age curve analysis, 1990-2021. (B) Period relative risk analysis curves, 1990-2021.

Using an age–period–cohort (APC) model, we first examined age effects on TBL deaths attributable to low fruit intake across SDI regions and globally. Cross-sectional age curves showed a consistent increase in TBL death burden with age in all regions, with a marked acceleration after age 60 (Figure 3A). Longitudinal age profiles confirmed this age-dependent rise (Figure S7A—see online supplementary material for a color version of this figure).

We then evaluated temporal trends using period effects from the APC model. Period relative risks declined in most regions, except in medium- and high-SDI regions, where relative risks increased after 2010. The most pronounced decline was observed in high-SDI regions (Figure 3B). Consistently, fitted temporal trend analyses indicated that TBL deaths increased after 2010 only in medium- and high-SDI regions, while other regions showed steady year-on-year declines (Figure S7B—see online supplementary material for a color version of this figure).

### Decomposition analysis of the burden of TBL associated with low-fruit diets globally and across SDI regions (1990-2021)

**Figure 4:**
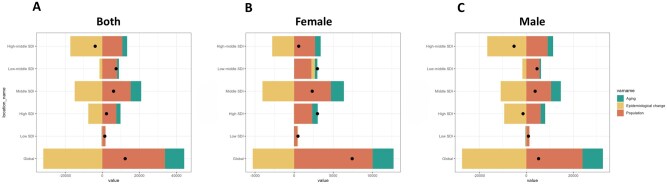
Decomposition analysis of the burden of TBL associated with low-fruit diets globally and across SDI regions from 1990 to 2021. (A) Both. (B) Female. (C) Male.

We conducted a decomposition analysis to quantify the relative contributions of aging, population growth, and demographically adjusted epidemiological changes to TBL deaths attributable to low-fruit diets across five SDI regions and globally. From 1990 to 2021, TBL deaths declined in high and high-middle SDI regions, mainly driven by epidemiological changes (Figure 4A). In contrast, burdens increased in the other regions and globally, with population growth being the primary driver. Aging reduced TBL deaths in low-SDI regions but increased them elsewhere (Figure 4A).

Gender-stratified analyses revealed that in females, TBL deaths rose in all SDI regions and globally, primarily due to population growth. Aging decreased the burden in low-SDI regions but increased it in others, while epidemiological changes contributed positively only in low-middle and low-SDI regions (Figure 4B).

In males, deaths declined in high and high-middle SDI regions, again largely attributable to epidemiological changes (Figure 4C). Similar to females, aging reduced the burden in low-SDI regions but had positive effects in other regions (Figure 4C).

### Potential for improvement in the burden of TBL associated with low-fruit diets across SDI regions

Using GBD data from 1990 to 2021, we systematically evaluated the burden of TBL attributable to low fruit intake and its potential for improvement across countries at different developmental levels. By integrating ASMR with SDI analyses, we identified countries with the greatest potential for reduction. The top 15 countries, ranked by effective variation range (104.38-33.09), included Mongolia, Lesotho, Zimbabwe, Cambodia, Zambia, Botswana, Timor-Leste, South Africa, Rwanda, Uganda, Burundi, Malawi, Cameroon, and Mali. Most of these countries had low SDI values (<0.5).

Notably, several high-SDI countries (>0.85), including the United Kingdom, Lithuania, Japan, Iceland, and Germany, also exhibited substantial improvement potential. These findings indicate that socioeconomic development alone does not fully determine diet-related disease burden, highlighting the critical role of targeted public health policies and dietary interventions (Figure S8A, B—see online supplementary material for a color version of this figure).

### Projection of low-fruit diet-related TBL burden to 2050

**Figure 5:**
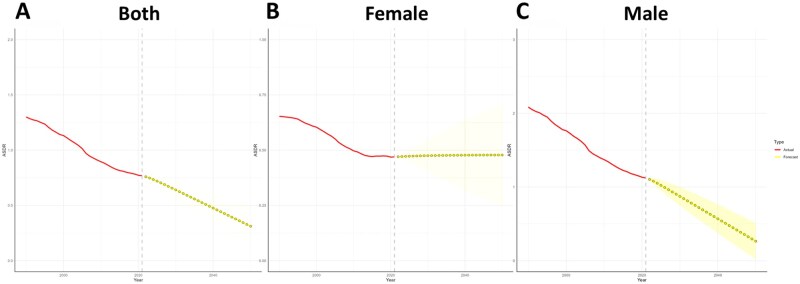
Projections of TBL mortality associated with low-fruit diets through 2025. (A) Both. (B) Female. (C) Male.

We employed an autoregressive integrated moving average (ARIMA) model to project the ASMR of TBL attributable to low-fruit diets up to 2050. Globally, the ASMR for TBL is projected to decline to 0.311 by 2050, indicating a clear downward trend (Figure 5A). However, this trend varied significantly by sex. For females, the ASMR is expected to remain stable at the 2021 level (0.478) by 2050, showing no significant change (Figure 5B). In contrast, the decline in ASMR was particularly pronounced among males, with a projected reduction to 0.265 by 2050 (Figure 5C).

### Causal protective effect of fruit intake on lung cancer: a MR meta-analysis

**Figure 6:**
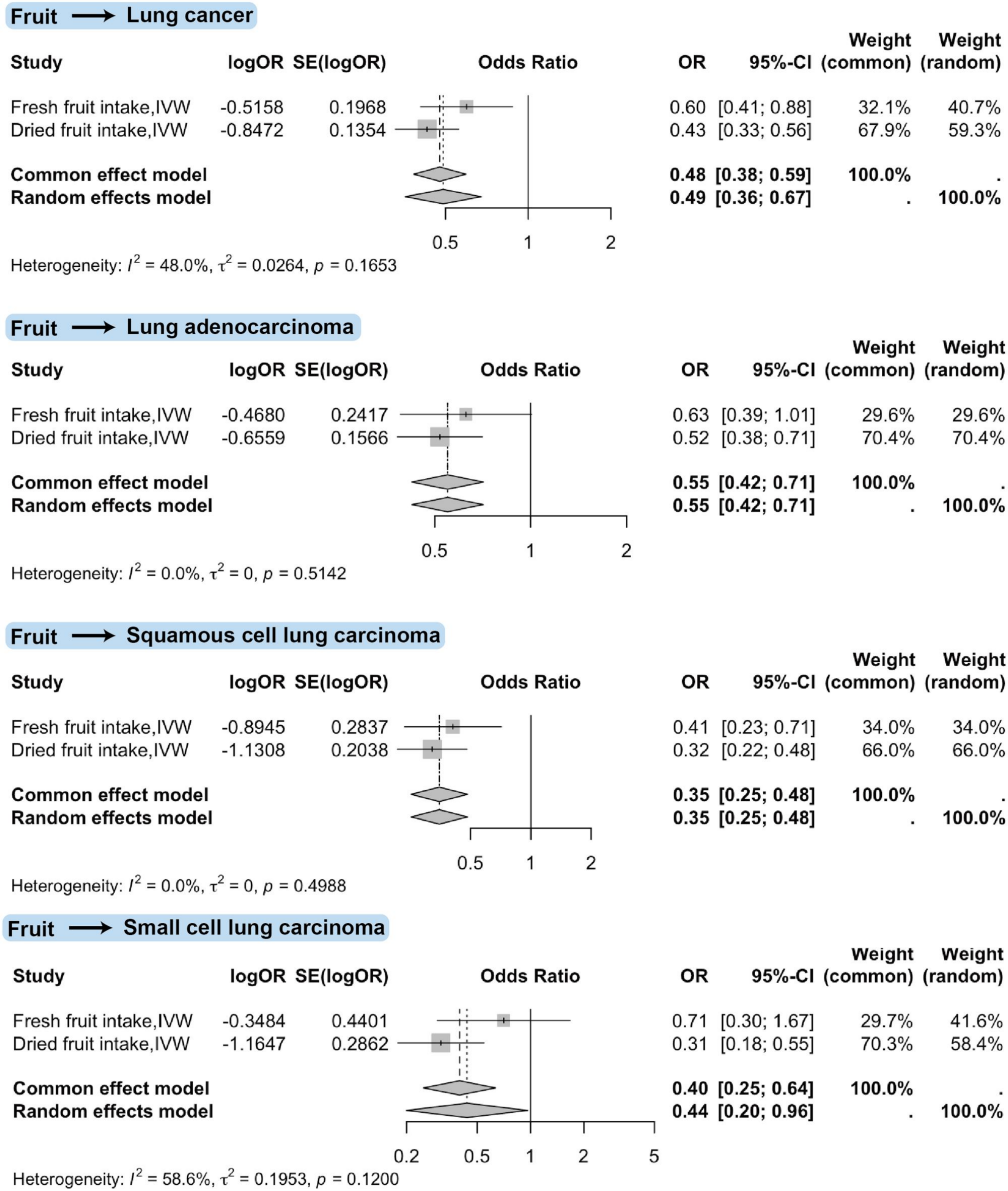
Meta-analysis of inverse variance weighted (IVW) Mendelian randomization estimates for the association between genetically predicted fruit intake and risk of lung cancer and its major subtypes.

To assess the potential causal association between fruit intake and lung cancer risk, we performed two-sample MR using summary-level data. The inverse variance weighted (IVW) method was the primary estimator, with weighted median, MR-Egger, simple mode, and weighted mode used for sensitivity analyses. Genetically predicted higher fruit intake was associated with reduced overall lung cancer risk (IVW OR = 0.48, 95% CI: 0.38-0.59) (Figure 6).

Subtype analyses showed inverse associations for adenocarcinoma (OR = 0.55, 95% CI: 0.42-0.71), squamous cell carcinoma (SCC) (OR = 0.35, 95% CI: 0.25-0.48), and small-cell carcinoma (OR = 0.40, 95% CI: 0.25-0.64). Moderate heterogeneity was observed for overall lung cancer and small-cell carcinoma (I^2^ = 48.0% and 58.6%), whereas other subtypes showed minimal heterogeneity. Dried fruit intake exhibited a relatively stronger inverse association.

MR-Egger estimates were non-significant for several subtypes, indicating potential sensitivity to directional pleiotropy. These findings should therefore be interpreted cautiously, representing suggestive rather than definitive causal evidence. Full MR and heterogeneity results are provided in Table S3 (see online supplementary material).

### Data overview of a real-world questionnaire survey on lung cancer

Baseline characteristics are summarized in Table S4 (see online supplementary material). Preliminary analyses showed significant differences in total fruit intake by lung cancer status. Patients with lung cancer generally consumed less fruit, with intake peaking at 250-500 g/day, whereas non-cancer participants showed higher and more widely distributed intake, peaking at 500-750 g/day, with a substantial proportion consuming >1000 g/day (Figure S9A—see online supplementary material for a color version of this figure).

Regarding BMI, most lung cancer cases were within the normal range (20-25), with fewer individuals classified as obese (BMI > 30), while overweight and obesity were more prevalent among non-cases (Figure S9B—see online supplementary material for a color version of this figure). This pattern may reflect disease-related weight loss among cancer patients.

Further analyses demonstrated significant associations between fruit intake, lung cancer risk, and sociodemographic factors. Higher fruit intake was associated with lower lung cancer risk (Figure S9C—see online supplementary material for a color version of this figure) and was more common among non-smokers, females, urban residents, and individuals with higher income and education (Figure S9D-H—see online supplementary material for a color version of this figure). Conversely, lower intake was more prevalent among lung cancer patients, smokers, males, rural residents, and those with lower socioeconomic status.

Together, these findings suggest that insufficient fruit intake is linked not only to increased lung cancer risk but also to broader social disadvantage, highlighting the importance of dietary interventions in public health strategies.

### SHAP analysis demonstrating the relative importance of lung cancer risk factors

To better control for confounders and assess the impact of high fruit intake on lung cancer incidence, we performed SHAP analysis. Age and education emerged as the strongest predictors, followed by fruit intake quantiles. Smoking, income, alcohol consumption, and sleep quality made moderate contributions, while BMI, marital status, gender, and residence were less influential (Figure S10A—see online supplementary material for a color version of this figure). Effect directions showed that smoking and older age increased lung cancer risk, whereas higher fruit intake and education were protective (Figure S10B—see online supplementary material for a color version of this figure).

### Interrelationships between fruit intake and non-dietary risk factors

Univariate analysis showed significant differences in household income (*P* < .001), smoking status (*P* < .001), gender (*P* = .018), education level (*P* = .002), and residential location (*P* = .013) across fruit intake quantiles, whereas alcohol use, age, BMI, and marital status showed no differences (Table S5—see online supplementary material). Spearman analysis further indicated that income (ρ = 0.22, *P* < .001) and education (ρ = 0.19, *P* < .001) were positively correlated with fruit intake, while smoking (ρ = −0.18, *P* < 0.001), rural residence (ρ = −0.12, *P* = 0.002), and male gender (ρ = −0.12, *P* = 0.003) were negatively correlated (Table S6—see online supplementary material).

In multivariable ordered logistic regression, higher household income remained significantly associated with greater fruit intake (OR = 1.50, 95% CI: 1.20-1.86, *P* < .001), while smoking predicted lower intake (OR = 0.63, 95% CI: 0.42-0.94, *P* = .022). Other variables (gender, education, BMI, residence, sleep, alcohol use, and age) showed no independent effects (Figure S11; Table S7—see online supplementary material). These findings suggest that socioeconomic and behavioral factors—particularly income and smoking—are key determinants of fruit intake, while other demographic influences appear limited.

### Non-dietary risk index (NDRI) constructed from non-fruit factors

Because non-dietary covariates were intercorrelated and potentially subject to multicollinearity (Figure S12A—see online supplementary material for a color version of this figure), we constructed a composite Non-Diet Risk Index (NDRI) using principal component analysis (PCA), incorporating age, sex, marital status, education, residence, income, BMI, sleep quality, smoking, and alcohol use. The scree plot indicated that PC1 explained the largest proportion of variance (20.4%), supporting its use as the NDRI component (Figure S12B—see online supplementary material for a color version of this figure).

**Figure 7:**
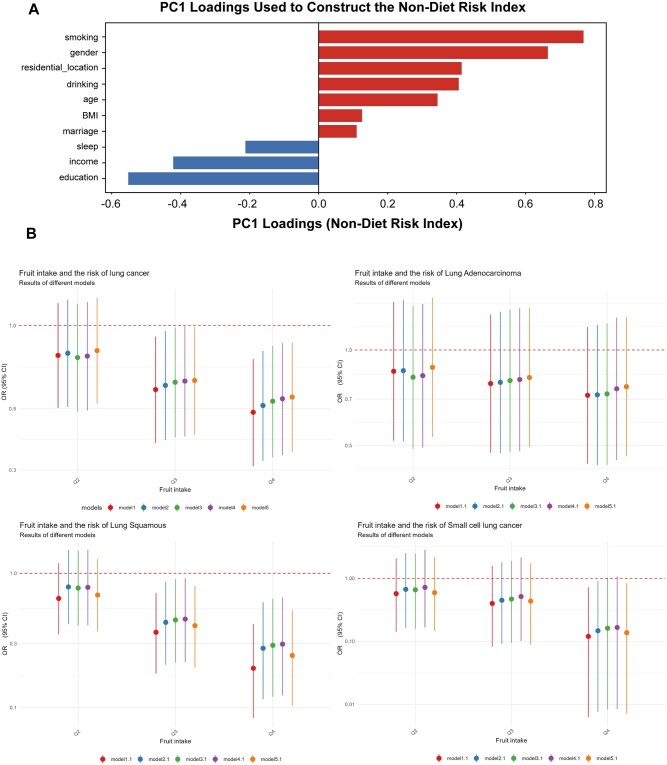
High fruit intake is a protective factor against lung cancer. (A) PC1 loadings used to construct the Non-Diet Risk Index (NDRI). (B) Fruit intake and the risk of lung cancer, adenocarcinoma, squamous and small cell lung cancer.

PC1 loadings showed positive contributions from smoking (0.77), male sex (0.66), alcohol use (0.41), rural residence (0.41), age (0.34), and BMI (0.13), while education (−0.55), income (−0.42), and sleep quality (−0.21) loaded negatively, indicating protective effects (Figure 7A; Figure S12C—see online supplementary material for a color version of this figure). The full loading matrix confirmed PC1 as the principal risk dimension (Table S8—see online supplementary material), and individual NDRI scores followed a continuous distribution (Table S9—see online supplementary material).

In logistic regression, higher NDRI was significantly associated with increased lung cancer risk (OR = 1.31, 95% CI: 1.17-1.46, *P* < .001), corresponding to approximately 30% higher risk per unit increase (Table S10—see online supplementary material). These findings support the use of NDRI as a composite adjustment variable in subsequent models.

### High fruit intake independently reduces lung cancer risk after NDRI adjustment

According to World Health Organization standards, we define each serving of fruit as 80 g. Daily intake for each fruit type is estimated by multiplying the specified serving size by the frequency of daily consumption. Our calculations reveal that in the Chinese hospital cohort, Q1 (low intake group) is defined as individuals consuming less than 30 g of fruit per day. Q2 represents the group with fruit intake of 30 g≤ fruit intake < 80 g, Q3 represents the group with fruit intake of 80 g≤ fruit intake < 180 g, and Q4 represents the group with fruit intake ≥ 180 g.

In multivariable logistic regression analyses adjusting for all confounders, higher fruit intake remained significantly protective against lung cancer. Compared with the lowest quartile (Q1), individuals in the highest quartile (Q4) had a consistently reduced risk across Models 1–5, with ORs of 0.485 (95% CI: 0.310-0.756, *P* = .001), 0.513 (95% CI: 0.324-0.809, *P* = .004), 0.532 (95% CI: 0.334-0.844, *P* = .008), 0.543 (95% CI: 0.340-0.863, *P* = .010), and 0.551 (95% CI: 0.349-0.868, *P* = .011), respectively (Figure 7; Table S11—see online supplementary material).

The third quartile (Q3) was also significantly associated with lower lung cancer risk (OR range: 0.587-0.633; all *P* < 0.05), whereas the second quartile (Q2) showed no statistically significant difference compared with Q1 (*P* > 0.05). Nevertheless, the graded reduction in ORs across quartiles suggests a dose–response relationship (Figure 7B; Table S11—see online supplementary material).

In fully adjusted models, smoking (OR range: 1.66-1.68, *P* < .05) and age (OR range: 2.05-2.18, *P* < .001) were significant risk factors. NDRI was independently associated with increased lung cancer risk (OR = 1.25, *P* < .01) (Figure S13—see online supplementary material for a color version of this figure). Sex, alcohol consumption, BMI, and sleep quality were not independently associated with lung cancer risk. Overall, the inverse association between fruit intake and lung cancer remained robust across sequential adjustment models, supporting a protective effect of higher fruit consumption.

To further investigate the relationship between fruit intake and the pathological subtypes of lung cancer, we conducted a subgroup analysis involving 295 patients with lung cancer, including 199 cases of adenocarcinoma, 82 cases of SCC, and 14 cases of small cell lung cancer (SCLC). The subgroup analysis indicated that higher fruit consumption was associated with a modest protective trend for lung adenocarcinoma (OR = 0.72, 95% CI: 0.44-1.18, *P* = .194), while significant protective effects were observed for SCC (OR = 0.244, 95% CI: 0.103-0.531, *P* < .001) and SCLC (OR = 0.12, 95% CI: 0.006-0.723, *P* = 0.052)( Tables S12-S14—see online supplementary material). These findings are generally consistent with the MR results.

### Effects of individual fruit types on lung cancer

To refine dietary guidance for lung cancer prevention, we examined fruit intake at both category and individual levels. Overall intake was higher in the non-lung cancer group. At the category level, citrus fruits (*P* = .007), tropical fruits (*P* = .005), and berries (*P* = .028) showed significant differences, whereas pome fruits, stone fruits, and melons displayed similar but nonsignificant trends (Figure S14A, B; Table S15—see online supplementary material).

At the individual level, grapes, grapefruit, bananas, and oranges were consumed significantly more in the non-lung cancer group (all *P* < .05), while apples and pears showed no significant differences (Figure S14C, D; Table S16—see online supplementary material). Effect size analysis identified oranges, grapes, and bananas as the largest contributors to intake differences. Overall, these findings are consistent with the multivariable regression results, indicating that higher consumption of citrus, tropical fruits, berries, grapes, grapefruit, bananas, and oranges is associated with non-lung cancer status, suggesting potential preventive relevance.

## Discussion

This study employed a triangulated analytical framework integrating GBD estimates, MR, and a hospital-based dietary validation study to explore the association between fruit intake and lung cancer risk. By merging causal inference, ecological burden estimation, and real-world observation, we identified convergent evidence that low fruit intake may increase lung cancer risk. This integrative approach highlights both the potential protective influence of fruit consumption and the multifactorial nature of diet-cancer interactions shaped by genetics, environment, and socioeconomic context.

We firstly analyzed GBD data to evaluate how insufficient fruit intake contributes to lung cancer burden across time and regions. This approach allows assessment of long-term trends and regional heterogeneity that individual-level studies cannot capture. Our analysis revealed a paradox: although age-standardized mortality and DALY rates attributable to low fruit intake declined, absolute numbers continued to rise, largely driven by population growth and aging. This pattern has also been observed in dietary risk factors for cardiovascular diseases and other cancers,^10–12^ indicating that demographic transitions can obscure progress in standardized rates. Importantly, the burden was heaviest in South and East Asia, with Sub-Saharan Africa showing increasing standardized rates, underscoring regional inequalities. However, GBD estimates are based on modeled dietary data that may be limited in low-income regions and cannot effectively separate correlated exposures such as vegetable or meat intake.

MR leverages germline genetic variants as instrumental variables to infer potential causal relationships between exposures and outcomes. By minimizing confounding and reverse causation, MR strengthens causal inference compared with conventional observational approaches.^13^ It has been increasingly applied to assess the causal impact of lifestyle and environmental factors, such as smoking, diet, and metabolic traits on chronic diseases, providing more robust evidence to guide prevention strategies and mechanistic understanding.^14^^,^^15^ In this study, MR analyses suggested that higher genetically predicted fruit intake was associated with lower risks of squamous and small-cell carcinoma subtypes. However, some sensitivity analyses revealed inconsistencies, indicating that the findings represent potential causal evidence. The average F-statistic exceeded 10, confirming sufficient instrument strength, yet the use of European GWAS data limits generalizability to non-European populations, including our Chinese cohort. Therefore, the interpretation should remain cautious. Importantly, the triangulation framework adopted in this study does not rely on strict population equivalence but rather on methodological complementarity. The MR component provides genetically anchored causal inference, whereas the hospital-based cohort offers population-specific observational validation. Although cross-ancestry differences in allele frequency, linkage disequilibrium structure, and gene–environment interactions may influence effect magnitude, the consistency in directionality across analytical approaches strengthens inference at the conceptual level. Considering the variability in genetic architecture and dietary patterns across regions, future multi-ancestry MR studies are needed to validate and extend these associations.

Recognizing that both GBD and MR rely on indirect measures, we conducted a hospital-based dietary survey to provide individual-level validation. After adjusting for key confounders—including smoking, alcohol intake, BMI, sleep quality, and socioeconomic status—higher fruit intake remained independently protective against lung cancer. Although smoking is the dominant established risk factor for lung cancer, the NDRI was designed to capture the aggregated background risk arising from correlated non-dietary characteristics rather than ranking causal importance. Variables such as sleep quality were included because they reflect broader lifestyle and socioeconomic contexts and shared variance with major behavioral risk factors, thereby improving confounder adjustment and model stability. The protective effect persisted across all models, with age and smoking emerging as the strongest risk factors. Subgroup analysis by histological subtype showed that fruit intake was significantly protective for SCC and SCLC, but only a non‑significant protective trend was observed for adenocarcinoma. The stronger effects for SCC and SCLC may reflect differences in carcinogenic pathways: SCC is closely linked to smoking‑induced oxidative stress and epithelial injury, while SCLC is characterized by high proliferative activity.^16^ Antioxidants abundant in fruit (eg, vitamin C, carotenoids, polyphenols) can neutralize free radicals, mitigate DNA oxidative damage, and suppress inflammation, potentially exerting greater protective effects in these contexts.^17–19^ The directionally consistent trend for adenocarcinoma suggests mechanistic differences, possibly involving driver mutations such as EGFR that may respond differently to dietary antioxidants.^20^ These findings align with our MR results, and their methodological convergence strengthens the evidence for a subtype‑specific protective effect of fruit, particularly for SCC.

Importantly, subgroup analyses showed that citrus, tropical fruits, and berries were most strongly associated with reduced risk, while grapes, bananas, and oranges contributed the largest effect sizes among individual fruits. These findings extend previous large cohort studies by providing fruit-specific evidence and linking dietary patterns with social determinants: higher fruit intake was positively associated with income and education but negatively associated with smoking and male sex. Based on the above research findings, we recommend establishing a tiered dietary intake target for clinical translation: a daily intake of 30 g should serve as the mandatory intervention threshold for risk warning, while 200-350 g (or at least 180 g) should be the core recommended target to achieve. Individuals should be encouraged to strive for the ideal intake level of 300-350 g to obtain optimal health benefits.

This study has several limitations. First, the genetic instruments were derived from European populations, which may limit generalizability to Asians due to ancestry-specific differences. Second, despite the use of multivariate models and sensitivity analyses using MR, the potential for residual pleiotropy and unmeasured confounding cannot be fully excluded. While the present analysis indicates a protective association, the magnitude of this effect may vary across populations. Third, the study’s projections for future declines in age-standardized lung cancer mortality rates are primarily based on historical trends over the past three decades, assuming that current dietary, economic, and socio-demographic patterns will largely persist. However, fruit consumption and its health implications may be significantly influenced by future factors such as food security conditions, economic fluctuations, agricultural policies, and global public health initiatives. Therefore, these projections should be viewed as scenario analyses under current trajectories, offering reference value for future public health planning and policy development. They require cautious interpretation and dynamic assessment within the context of evolving socioeconomic conditions. Fourth, in the histological subtype subgroup analysis, the relatively small sample size for SCLC limits the stability of the estimates, which should be confirmed in larger prospective cohorts. Future research should incorporate more granular dietary composition data, biomarker measurements, and tumor molecular profiling to elucidate the specific fruit components and molecular mechanisms underlying subtype-specific protective effects. Finally, the hospital cohort was moderate in size and relied on self-reported diet, which may introduce recall bias. Future multi-ancestry MR and large-scale prospective studies are warranted to validate these findings.

Taken together, these complementary approaches provide consistent evidence that insufficient fruit intake increases lung cancer risk. GBD highlights the long-term global burden, MR indicates a possible protective genetic association, and the hospital-based data validates individual-level relevance. The integration of these findings underscores the triangulation rationale—using different data sources to strengthen inference while acknowledging each method’s unique limitations, underscoring dietary modification as a promising strategy to complement tobacco control, air quality improvement, and early detection. Collectively, our results establish low fruit intake as a modifiable determinant of lung cancer and support the incorporation of nutritional assessment into precision prevention strategies. Looking forward, further research and coordinated policy efforts will be essential to translate these insights into effective interventions that help reduce the global lung cancer burden.

## Conclusion

Together, GBD, MR, and our hospital-based survey provide consistent evidence that insufficient fruit intake increases lung cancer risk. MR suggests causality, GBD defines the global and regional burden, and real-world data highlight fruit-specific and population-level effects. Despite methodological limitations, their integration strengthens inference and supports dietary modification as a complement to smoking cessation, air quality control, and early detection. Future work should validate these findings in diverse populations and assess fruit-promotion strategies through pragmatic interventions.

## Supplementary Material

oyag069_Supplementary_Data

## Data Availability

The data used and/or analyzed in the current study are available from the corresponding authors on request.

## Abbreviations

AAPC: average annual percentage change
AIC: Akaike information criterion
APC: annual percentage change
ARIMA: autoregressive integrated moving average models
ASDR: age-standardized DALY rates
ASMR: age-standardized mortality rates
ASR: age-standardized rate
BIC: Bayesian information criterion
BMI: body mass index
Cis: confidence intervals
DALYs: disability-adjusted life years
EAPC: estimated annual percentage change
FFQ: food frequency questionnaire
GBD: Global Burden of Disease
ICC: intraclass correlation coefficient
IVW: inverse variance weighted
MR: Mendelian randomization
NDRI: Non-Diet Risk Index
OR: odds ratio
PCA: principal component analysis
RR: relative risk
SCC: squamous cell carcinoma
SCLC: small cell lung cancer
SDI: socio-demographic index
SHAP: SHapley Additive exPlanations
TBL: tracheal, bronchial, and lung (TBL) cancers
