# Supplementary material for: Triangulating associations between fruit intake and lung cancer risk: evidence from GBD estimates, Mendelian randomization, and real-world validation
Source: Oncologist. 2026 Feb 27;31(7):oyag069. doi: 10.1093/oncolo/oyag069 (PMC13329070; doi:10.1093/oncolo/oyag069)
Supplement: oyag069_Supplementary_Data [file oyag069_supplementary_data.zip › Legends of Supplementary Figure&Table.docx]

**Supplementary Figure 1 The screening flowchart of the hospital-based cross-sectional study.**

**Supplementary Figure 2 204 National EAPCs.**

(A) EAPC(ASMR). (B) EAPC (ASDR).

**Supplementary Figure 3** **Association of low-fruit diet-associated TBL burden with SDI.**

(A) Fitting analysis of low-fruit diet-associated TBL ASMR to SDI levels in 204 GBD countries. (B) Fitting analysis of low-fruit diet-associated TBL ASMR to SDI levels in 21 GBD regions.

**Supplementary Figure 4 Age and sex specific changes in the burden of TBL associated with a low-fruit diet.**

(A) Number of deaths by sex in different age groups in 2021. (B) Trends in deaths by sex with age in 2021. (C) Trends in the number of deaths by sex over time, 1990-2021. (D) Trends in the number of deaths by sex over time, 1990-2021.

**Supplementary Figure 5 Age and sex specific changes in TBL burden associated with low-fruit diets.**

(A) Number of DALYs by sex in different age groups in 2021. (B) Trends in rates of DALYs by sex with age in 2021. (C) Trends in the number of DALYs by sex over time, 1990-2021. (D) Trends in rates of DALYs by sex over time, 1990-2021.

**Supplementary Figure 6** **Rates of DALYs in different age groups and in different SDI regions for the burden of TBL associated with a low-fruit diet.**

(A) Trends in DALYs rates across SDI regions in different age groups, 1990-2021. (B) Trends in EAPC of DALYs rates across SDI regions in different age groups, 1990-2021.

**Supplementary Figure 7** **Age-Period-Cohort Effects on TBL Burden Associated with Low-Fruit Diets.**

(A) Longitudinal age-curve analysis of trends, 1990-2021. (B) Time-trend analysis curves, 1990-2021.

**Supplementary Figure 8 Potential for Improvement in TBL Burden Associated with Low-Fruit Diets.**

(A) Frontier analysis of TBL burden associated with low-fruit diets, 1990-2021. (B) Frontier analysis of TBL burden associated with low-fruit diets in 2021.

**Supplementary Figure 9 Distribution and Association of Lung Cancer Status with Fruit Intake, BMI, and Demographic Characteristics.**

(A) Distribution of total fruit intake across lung cancer status groups (B) Distribution of lung cancer status groups and body mass index (BMI) (C) Lung cancer status (D) Smoking (E) Income level (F) Gender (G) Educational attainment (H) Association analysis between place of residence and quartile of fruit intake

**Supplementary Figure 10 Multifactorial Analysis of Lung Cancer Risk Factors**

(A)Standardized mean difference (B) Multivariate-adjusted regression coefficient

**Supplementary Figure 11 Effects of Different Confounding Factors on Fruit Intake**

**Supplementary Figure 12 NDRI Model Construction**

(A)Heatmap of Correlation Analysis Among Risk Factors (B) Scree plot of principal component analysis. (C) Heatmap of loadings on PC1 (NDRI).

**Supplementary Figure 13 Logistic Regression Model Heatmap**

**Supplementary Figure 14 Differences in average intake between non-lung cancer and lung cancer populations across different fruit categories and specific fruit varieties**

(A) Comparison of intake levels for major fruit categories between the two groups (B) Differences in mean values across major fruit categories (C) Comparison of intake levels for specific fruit varieties between the two groups (D) Differences in mean values across specific fruit varieties

**Supplementary Table 1 TBL burden associated with low-fruit diet and its temporal trends, globally and in different regions of SDI, 1990 and 2021**

**Supplementary Table 2** **TBL burden associated with low-fruit diets and its temporal trends in 21 GBD regions, 1990 and 2021**

**Supplementary Table 3 Mendelian randomization estimates and heterogeneity statistics for the association between fruit intake and lung cancer risk.**

**Supplementary Table 4 Baseline Characteristics of Participants**

**Supplementary Table 5 Univariate Analysis of Confounding Factors on Fruit Intake**

**Supplementary Table 6 Spearman's analysis of the Influence of Confounding Factors on Fruit Intake**

**Supplementary Table 7 Multivariate Ordered Logistic Regression Analysis of Confounding Factors' Effects on Fruit Intake**

**Supplementary Table 8 PCA overall loadings**

**Supplementary Table 9 Non-Diet Risk Index (NDRI) scores**

**Supplementary Table 10 Logistic Regression Model of NDRI and Lung Cancer Risk**

**Supplementary Table 11 Logistic regression modeling of the association between fruit intake and lung cancer**

**Supplement Table 12 Logistic regression modeling of the association between fruit intake and lung adenocarcinoma**

**Supplement Table 13 Logistic regression modeling of the association between fruit intake and squamous cell carcinoma**

**Supplement Table 14 Logistic regression modeling of the association between fruit intake and small cell lung cancer**

**Supplementary Table 15 The Effect of Fruit Categories on Lung Cancer Risk**

**Supplementary Table 16 The Effect of Fruit Types on Lung Cancer Risk**
