# Supplementary material for: Triangulating associations between fruit intake and lung cancer risk: evidence from GBD estimates, Mendelian randomization, and real-world validation
Source: Oncologist. 2026 Feb 27;31(7):oyag069. doi: 10.1093/oncolo/oyag069 (PMC13329070; doi:10.1093/oncolo/oyag069)
Supplement: oyag069_Supplementary_Data [file oyag069_supplementary_data.zip › Supplementary Material.docx]

# **2. Materials and Methods**

**2.1 Mendelian Randomization**

Two-sample Mendelian randomization (MR) analyses were performed to investigate the causal relationship between fruit intake and lung cancer risk. Genetic instruments for fresh fruit intake (ukb-b-3881) and dried fruit intake (ukb-b-16576) were obtained from the UK Biobank via the MRC IEU Open GWAS database. To increase statistical power for traits with few genome-wide significant hits, we adopted a relaxed selection threshold (p < 5 × 10^-6^), followed by clumping (r^2^ < 0.01, 10,000 kb window). The mean F-statistics exceeded 10, confirming sufficient instrument strength. Outcome GWAS data were derived from large European cohorts, including overall lung cancer (ebi-a-GCST004748), lung adenocarcinoma (ebi-a-GCST004744), squamous cell carcinoma (ebi-a-GCST004750), and small cell lung carcinoma (ebi-a-GCST004746). The primary analysis used the inverse variance weighted (IVW) method under a fixed-effect model. Sensitivity analyses included MR-Egger, weighted median, simple mode, and weighted mode. Horizontal pleiotropy and heterogeneity were assessed using the MR-Egger intercept and Cochran’s Q statistic, respectively. All MR analyses were conducted using the Two-Sample MR and MRPRESSO packages in R, and the meta-analysis of IVW results was performed using the meta package in R.

**2.2 Data Collection**

Data for the GBD component were obtained from the Global Burden of Disease (GBD) 2021 database, providing estimates of deaths, age-standardised mortality rate (ASMR), disability-adjusted life years (DALYs), and age-standardised DALY rate (ASDR) for tracheal, bronchial, and lung cancers attributable to dietary risks. Estimates were stratified by sex, five-year age group (25–29 to≥95 years), and socio-demographic index (SDI). Because GBD data integrate wealth and education indicators within SDI, these confounding factors were indirectly adjusted at the ecological level.

**2.3 Socio-demographic Index (SDI)**

Countries and regions were categorized into five groups based on the socio-demographic index (SDI): high SDI (>0.81), medium-high SDI (0.70–0.81), medium SDI (0.61–0.70), medium-low SDI (0.46–0.61), and low SDI (<0.46). The SDI is a composite index that integrates three key indicators: per capita income, total fertility rate for individuals under 25 years of age, and average education level for individuals aged 15 years and older. The SDI ranges from 0 to 1, with higher values indicating higher socioeconomic development.

**2.4 Study Population**

The hospital-based cross-sectional component was conducted at the First Hospital of China Medical University (from December 2024 to September 2025). Adults (≥18 years) with pathologically confirmed benign or malignant lung nodules were enrolled. After exclusions for missing data or extrapulmonary metastases, 641 participants remained (346 benign, 295 malignant) (Supplementary Figure 1). Ethical approval was granted (Ethics Review [2024]-591-2), and all participants provided written informed consent.

**2.5 Dietary assessment**

Dietary intake was assessed using a validated Food Frequency Questionnaire (FFQ) developed by Professor Qijun Wu’s team(1).This tool, adapted from the Northeast China Cohort Study, demonstrated high reliability (intraclass correlation coefficient [ICC] = 0.72) and validity (correlation coefficient r = 0.61 against 24-hour dietary recalls) in prior studies(2, 3).Trained investigators administered the FFQ through face-to-face interviews, capturing participants’ habitual dietary intake over the preceding year. For each food item, respondents reported consumption frequency using standardized response options: (i.e., “≥2 times per day” “once per day,” “4-6 times per week,” “2-3 times per week,” “2-3 times per week,” “once per week,” “1-6 times per month,” and “1-3 times per month”). times per week“, ”2-3 times per week“, ”once per week“, ”1-3 times per month "), we defined each portion of fruit as 80g according to the WHO standard, and multiplied the specified portion size by the frequency of consumption per day to estimate the daily consumption of each fruit(3).

**2.6 Statistical Analysis**

**2.6.1 Estimated annual percentage change (EAPC)**

To assess trends in age-standardized rates (ASRs) from 1990 to 2021, we employed the estimated annual percentage change (EAPC). The EAPC is a widely used statistical metric that quantifies the average annual rate of change in a specific indicator over time. It provides a measure of the percentage change in the indicator from one year to the next. The EAPC was calculated using the following linear regression model:

$$\ln(ASR)=\alpha+\beta x+\varepsilon$$

$$EAPC=100\times(\exp(\beta)-1)$$

In this model, x represents the calendar year, ε denotes the error term, and β indicates the direction and magnitude of the trend in the age-standardized rate (ASR). By applying a log transformation to the ASR, we can model the relative change in rates over time, ensuring that the trends follow a linear pattern. This method is widely adopted in epidemiological studies to estimate the percentage change in incidence or mortality rates over time (4-6). Using this model, the EAPC and its 95% confidence intervals (CIs) can be derived. Trends are interpreted as follows: A trend is classified as increasing if both the EAPC and its 95% CI are greater than 0, decreasing if both are less than 0, and stable if the 95% CI includes 0.

**2.6.2 Joinpoints: Time Trend Analysis**

Joinpoints regression is a time series analysis method used to identify points of trend change (joinpoints) in the data and segment the data into distinct trend phases(7). It optimizes the overall model by fitting segmented regression to each trend phase. The primary outcome indicators include the annual percentage change (APC), which reflects the year-to-year percentage change in the dependent variable, and the average annual percentage change (AAPC), which represents the average percentage change over the selected time period.

**2.6.3 Age-Period-Cohort Model (APC Model)**

The Age-Period-Cohort (APC) model is a statistical tool widely used in epidemiology to analyze and disentangle the three major effects influencing disease trends: age, period, and cohort effects(8). This model allows for the simultaneous interpretation of the impact of these three variables on the burden of TBL attributable to low-fruit diets. Data were divided into consecutive 5-year intervals from 1992 to 2021; data from 1990–1991 were excluded as they did not span a full 5-year interval. The age range was restricted to 25–84 years, with individuals older than 85 years excluded from the analysis. The reference group was defined as the average of the age, period, and cohort effects, and the relative risk (RR) for each age, period, and cohort was calculated as the independent risk compared to the reference group.

**2.6.4 Decomposition Analysis**

Decomposition analysis was employed to quantify the relative contributions of population growth, aging, and epidemiological changes to the burden of TBL attributable to low-fruit dietary factors. This analysis was conducted on a global scale and further stratified by socio-demographic index (SDI) regions to identify the main drivers of disease burden and their variations across different socioeconomic contexts. This approach not only enhances the understanding of the composition of the global TBL disease burden but also provides a critical foundation for targeted intervention strategies tailored to regions at different developmental levels.

**2.6.5 Autoregressive Integrated Moving Average (ARIMA) Model**

The ARIMA (AutoRegressive Integrated Moving Average) model is a widely used time series analysis method for forecasting future values(9). By integrating three components—autoregressive (AR), differencing (I), and moving average (MA)—the ARIMA model effectively captures trends and seasonal variations in time series data.

**2.6.6** **SHAP Analysis on Lung Cancer Risk Factors**

To preview the relative roles of demographic, lifestyle, and socioeconomic factors in lung cancer risk, we constructed a gradient boosting tree–based classification model (XGBoost) and applied the SHapley Additive exPlanations (SHAP) method to interpret the model outputs. SHAP, grounded in game theory, quantifies the marginal contribution of each variable across all possible feature combinations, providing a robust measure of direction and magnitude(10). We generated SHAP summary plots and feature importance bar plots to identify the most influential variables at the population level and to provide exploratory insights for subsequent analyses.

**2.6.7 Relationship Between Demographic Characteristics and Lifestyle Factors and Fruit Intake Percentiles**

To evaluate the associations between demographic and lifestyle characteristics and fruit intake quartiles, three complementary statistical methods were applied. First, categorical predictors (gender, marital status, smoking, drinking, and residential location) were examined using the Chi-square test of independence to assess differences in distribution across fruit intake quartiles (Q1–Q4). The variables were coded as follows: gender (0 = female, 1 = male); marital status (0 = unmarried, 1 = married, 2 = divorced, 3 = widowed); drinking status (0 = non-drinker, 1 = drinker); residential location (0 = urban, 1 = rural); and age group (0 = <65 years, 1 = ≥65 years). Second, ordinal or continuous predictors (such as BMI, education level, and household income) were analyzed using Spearman’s rank correlation to detect monotonic trends with fruit intake quartiles. Finally, a multivariable ordinal logistic regression model (proportional odds model) was fitted, with fruit intake quartile as the dependent variable and all covariates included simultaneously as independent variables. Odds ratios (ORs) with 95% confidence intervals (CIs) were calculated to assess independent associations. Model fit was evaluated using the log-likelihood, Akaike information criterion (AIC), and Bayesian information criterion (BIC). A two-sided p-value < 0.05 was considered statistically significant.

**2.6.8** **Non-Diet Risk Index (NDRI) Analysis in Lung Cancer Study**

To mitigate potential multicollinearity among non-diet covariates (age, gender, marital status, education, residential location, income, body mass index [BMI], sleep quality, smoking, and drinking), we applied principal component analysis (PCA) to construct a composite Non-Diet Risk Index (NDRI). All variables were standardized prior to analysis, and the first principal component (PC1) was extracted as the NDRI. PC1 loaded positively on smoking (0.37), drinking (0.36), male sex (0.35), older age (0.33), BMI (0.22), and rural residence (0.28), while education (-0.32), income (-0.31), and sleep quality (-0.15) showed negative loadings. PC1 explained 20.4% of the variance, and the first four components together explained 60.5%. The direction of PC1 was aligned with lung cancer status such that higher NDRI values indicated higher non-diet risk. Logistic regression was then performed with lung cancer as the dependent variable and NDRI as the independent variable, yielding odds ratios (ORs) and 95% confidence intervals (CIs).

**2.6.9 Logistic Regression**

Logistic regression was used to analyze the effect between fruit diet and lung cancer. The effect of fruit intake on lung cancer was explored and analyzed by transforming fruit intake into a categorical variable, i.e., it was expressed in quartiles (25%, 50%, and 75%), and the group with the lowest fruit intake (< 25%) was used as the control group. In order to control the influence of other confounding factors on the results, we used multiple regression analysis and established five models to explore the effect of fruit intake on lung cancer: model 1 did not treat any confounding factors; model 2 adjusted for age and gender; model 3 adjusted for age, gender, smoking, and alcohol consumption; model 4 adjusted for gender, age, smoking, alcohol consumption, Body Mass Index (BMI) and sleep quality; model 5 adjusted for NDRI.

**2.6.10 Software and Statistical Significance**

Joinpoints analysis was performed using the Joinpoints software, and all other statistical analyses were conducted using R version 4.3.2. Fruit intake data (both at the category and individual fruit levels) between the lung cancer and non-lung cancer groups were compared using a two-tailed Student’s t-test. Statistical significance was defined as a two-sided p-value of less than 0.05.

**Reference**

1. Li XY, Zhang YX, Wang XB, Nan YX, Wang DD, Sun MH, et al. Associations between dietary macronutrient quality and asthenozoospermia risk: a hospital-based case-control study. Food & function. 2024;15(12):6383–94.

2. Chang Q, Wu Q, Xia Y, Zhang H, Gao S, Zhang Y, et al. Cohort Profile: The Northeast China Biobank (NEC-Biobank). International journal of epidemiology. 2023;52(2):e125–e36.

3. Cui Q, Xia Y, Liu Y, Sun Y, Ye K, Li W, et al. Validity and reproducibility of a FFQ for assessing dietary intake among residents of northeast China: northeast cohort study of China. Br J Nutr. 2023;129(7):1252–65.

4. Feng H, Li Z, Zheng R. The global burden of chronic respiratory diseases attributable to tobacco from 1990 to 2021: a global burden of disease study 2021. BMC public health. 2025;25(1):456.

5. He M, Gu R, Huang X, Zhao A, Liu F, Zheng Y. Age-period-cohort analysis of gallbladder and biliary diseases epidemiological trends from 1990 to 2021 and forecasts for 2035: a systematic analysis from the global burden of disease study 2021. BMC gastroenterology. 2025;25(1):7.

6. Yang X, Fang Y, Chen H, Zhang T, Yin X, Man J, et al. Global, regional and national burden of anxiety disorders from 1990 to 2019: results from the Global Burden of Disease Study 2019. Epidemiology and psychiatric sciences. 2021;30:e36.

7. Noé L, Valtuille Z, Lanoy E, Katsahian S, Kaguelidou F. Evaluation of methods for joinpoint analysis of time series using simulated and real-world data. J Clin Epidemiol. 2025:111966.

8. Rosenberg PS, Check DP, Anderson WF. A web tool for age-period-cohort analysis of cancer incidence and mortality rates. Cancer Epidemiol Biomarkers Prev. 2014;23(11):2296–302.

9. Schaffer AL, Dobbins TA, Pearson SA. Interrupted time series analysis using autoregressive integrated moving average (ARIMA) models: a guide for evaluating large-scale health interventions. BMC Med Res Methodol. 2021;21(1):58.

10. Cao M, Zou J, Shi M, Zhao D, Liu C, Liu Y, et al. A promising therapeutic: Exosome-mediated mitochondrial transplantation. International Immunopharmacology. 2024;142:113104.
