# Supplementary material for: Triangulating associations between fruit intake and lung cancer risk: evidence from GBD estimates, Mendelian randomization, and real-world validation
Source: Oncologist. 2026 Feb 27;31(7):oyag069. doi: 10.1093/oncolo/oyag069 (PMC13329070; doi:10.1093/oncolo/oyag069)
Supplement: oyag069_Supplementary_Data [file oyag069_supplementary_data.zip › Supplementary Table 1.docx]

| **Supplementary Table 1 TBL burden associated with low-fruit diet and its temporal trends, globally and in different regions of SDI, 1990 and 2021** | | | | | | | | | | |
| --- | --- | --- | --- | --- | --- | --- | --- | --- | --- | --- |
| Location | Death | | | | DALY | | | | EAPC | |
|  | 1990 | | 2021 | | 1990 | | 2021 | |  |  |
|  | Cases No. (95%UI) | ASMR per 100 000 (95% UI) | Cases No. (95%UI) | ASMR per 100 000 (95% UI) | Cases No. (95%UI) | ASDR per 100 000 (95% UI) | Cases No. (95%UI) | ASDR per 100 000 (95% UI) | ASMR per 100 000 (95% UI) | ASDR per 100 000 (95% UI) |
| Global | 51621 (25770,75860) | 1.3 (0.65,1.91) | 66045 (34006,97033) | 0.77 (0.4,1.13) | 1435375 (721499,2119984) | 34.39 (17.24,50.7) | 1611267 (828054,2347369) | 18.46 (9.49,26.9) | -1.79  (-2.69, -0.87) | -2.11  (-3, -1.22) |
| SDI | | | | | | | | | | |
| Low SDI | 1410 (744,2181) | 0.62 (0.33,0.96) | 2800 (1383,3998) | 0.57 (0.28,0.82) | 41194 (21755,63800) | 16.45 (8.68,25.52) | 79513 (39179,113066) | 14.28 (7.06,20.35) | -0.06  (-1.13, 1.01) | -0.26  (-1.16, 0.65) |
| High SDI | 13872 (7057,20227) | 1.25 (0.64,1.82) | 16064 (8040,24167) | 0.73 (0.37,1.1) | 336402 (170224,487385) | 31.18 (15.77,45.16) | 322709 (163062,479679) | 16.18 (8.21,24.09) | -1.53  (-2.32, -0.73) | -1.9  (-2.65, -1.14) |
| Middle SDI | 14843 (7434,22198) | 1.44 (0.72,2.15) | 20847 (10804,30841) | 0.79 (0.41,1.17) | 435242 (214869,649545) | 37.98 (18.93,56.7) | 529166 (271428,776883) | 18.97 (9.76,27.83) | -2.22  (-3.09, -1.34) | -2.54  (-3.4, -1.67) |
| Low-middle SDI | 5795 (3056,8625) | 0.94 (0.5,1.41) | 12105 (6258,17015) | 0.84 (0.44,1.18) | 169941 (89659,252673) | 25.21 (13.29,37.46) | 339645 (176183,478078) | 21.96 (11.38,30.93) | -0.19  (-1.11, 0.74) | -0.26  (-1.16, 0.65) |
| High-middle SDI | 15653 (7765,23388) | 1.55 (0.77,2.31) | 14175 (7072,21347) | 0.71 (0.36,1.07) | 451238 (225373,672450) | 43.32 (21.63,64.65) | 338956 (167188,513037) | 17.17 (8.48,25.96) | -2.8  (-3.88, -1.7) | -3.3  (-4.36, -2.24) |
