# Supplementary material for: Triangulating associations between fruit intake and lung cancer risk: evidence from GBD estimates, Mendelian randomization, and real-world validation
Source: Oncologist. 2026 Feb 27;31(7):oyag069. doi: 10.1093/oncolo/oyag069 (PMC13329070; doi:10.1093/oncolo/oyag069)
Supplement: oyag069_Supplementary_Data [file oyag069_supplementary_data.zip › Supplementary Table 2.docx]

| **Supplementary Table 2 TBL burden associated with low-fruit diets and its temporal trends in 21 GBD regions, 1990 and 2021** | | | | | | | | | | |
| --- | --- | --- | --- | --- | --- | --- | --- | --- | --- | --- |
| Location | Death | | | | DALY | | | | EAPC | |
|  | 1990 | | 2021 | | 1990 | | 2021 | |  |  |
|  | Cases No. (95%UI) | ASMR per 100 000 (95% UI) | Cases No. (95%UI) | ASMR per 100 000 (95% UI) | Cases No. (95%UI) | ASDR per 100 000 (95% UI) | Cases No. (95%UI) | ASDR per 100 000 (95% UI) | ASMR per 100 000 (95% UI) | ASDR per 100 000 (95% UI) |
| Oceania | 26 (12,44) | 0.95 (0.44,1.63) | 57 (26,96) | 0.82 (0.38,1.36) | 762 (346,1301) | 23.56 (10.7,40.04) | 1643 (735,2810) | 19.92 (9.07,33.59) | -0.33  (-0.91, 0.25) | -0.37  (-0.92,  0.17) |
| Southeast Asia | 3462 (1762,5213) | 1.38 (0.7,2.07) | 4374 (2127,6612) | 0.68 (0.33,1.03) | 99020 (50497,149303) | 35.6 (18.14,53.73) | 118214 (57829,179100) | 16.87 (8.24,25.48) | -2.46  (-3.04 , -1.87) | -2.62  (-3.17 , -2.06) |
| East Asia | 17900 (8752,27451) | 2.12 (1.04,3.22) | 19553 (9989,30557) | 0.92 (0.47,1.44) | 520776 (255283,780491) | 54.78 (26.76,82.71) | 455284 (229549,716373) | 20.72 (10.48,32.5) | -3.06  (-3.61, -2.5) | -3.56  (-4.08 , -3.03) |
| Central Asia | 860 (438,1252) | 1.78 (0.9,2.59) | 381 (196,560) | 0.46 (0.24,0.68) | 26223 (13391,38103) | 52.05 (26.47,75.81) | 10854 (5572,15980) | 12.13 (6.24,17.81) | -4.7  (-5.66 ,  -3.73) | -5.04  (-6, -4.08) |
| Eastern Europe | 5642 (2928,8218) | 1.97 (1.02,2.87) | 2826 (1449,4147) | 0.8 (0.41,1.17) | 164166 (85551,238346) | 57.59 (30.06,83.5) | 72402 (37070,105901) | 21.24 (10.9,31.11) | -3.39  (-4.63 , -2.14) | -3.67  (-4.92 , -2.41) |
| High-income Asia Pacific | 2045 (1054,2953) | 1.04 (0.54,1.49) | 3789 (1927,5599) | 0.72 (0.36,1.06) | 47430 (24351,68657) | 23.25 (11.95,33.66) | 65117 (32978,96290) | 15.02 (7.65,22.08) | -1.05  (-1.89 , -0.2) | -1.2  (-2.03 , -0.37) |
| Central Europe | 2074 (1054,3002) | 1.37 (0.7,1.99) | 2224 (1136,3283) | 1 (0.51,1.47) | 58378 (29835,84085) | 38.55 (19.69,55.54) | 51738 (26283,76271) | 24.62 (12.5,36.26) | -0.74  (-1.61 , 0.14) | -1.12  (-1.98 ,  -0.25) |
| Australasia | 240 (120,351) | 1.01 (0.5,1.47) | 329 (169,488) | 0.59 (0.31,0.88) | 5698 (2861,8319) | 24.37 (12.22,35.55) | 6700 (3486,9909) | 13.06 (6.83,19.32) | -1.56  (-2 ,  -1.12) | -1.84  (-2.23 , -1.45) |
| Western Europe | 5340 (2699,7762) | 0.92 (0.47,1.34) | 5650 (2849,8259) | 0.6 (0.3,0.87) | 125376 (63353,181485) | 22.91 (11.58,33.11) | 116950 (59350,169417) | 13.97 (7.06,20.24) | -0.98  (-1.62 , -0.33) | -1.14  (-1.75 , -0.53) |
| High-income North America | 4798 (2457,7009) | 1.37 (0.7,2.01) | 4754 (2372,7308) | 0.7 (0.35,1.07) | 116403 (59213,170120) | 34.99 (17.78,50.97) | 98586 (49205,150590) | 15.31 (7.67,23.3) | -2.1  (-2.42 , -1.78) | -2.58  (-2.87 , -2.28) |
| Southern Latin America | 326 (164,476) | 0.7 (0.35,1.03) | 280 (142,421) | 0.32 (0.16,0.48) | 8791 (4446,12844) | 18.75 (9.49,27.41) | 6389 (3226,9597) | 7.47 (3.75,11.23) | -2.12  (-2.85 , -1.38) | -2.52  (-3.24 , -1.79) |
| Andean Latin America | 78 (40,122) | 0.4 (0.2,0.62) | 128 (62,200) | 0.22 (0.11,0.34) | 2085 (1061,3222) | 9.59 (4.86,14.87) | 3053 (1460,4756) | 5.06 (2.42,7.86) | -2.04  (-2.28 , -1.8) | -2.27  (-2.5, -2.05) |
| Caribbean | 124 (62,186) | 0.49 (0.24,0.72) | 167 (81,248) | 0.31 (0.15,0.46) | 3071 (1529,4606) | 11.61 (5.77,17.42) | 3954 (1896,5867) | 7.35 (3.52,10.89) | -1.28  (-1.78 , -0.76) | -1.36  (-1.85 , -0.88) |
| Central Latin America | 252 (127,366) | 0.32 (0.16,0.46) | 483 (244,711) | 0.2 (0.1,0.29) | 6687 (3362,9670) | 7.56 (3.8,10.97) | 11772 (5939,17378) | 4.62 (2.33,6.82) | -1.62  (-1.98 , -1.25) | -1.68  (-2.02 , -1.33) |
| North Africa and Middle East | 465 (228,726) | 0.28 (0.14,0.44) | 845 (425,1277) | 0.19 (0.1,0.29) | 13430 (6596,21038) | 7.25 (3.56,11.35) | 23506 (11719,35335) | 4.7 (2.36,7.1) | -1.08  (-1.95 , -0.2) | -1.26  (-2.09 , -0.41) |
| Tropical Latin America | 322 (163,475) | 0.36 (0.18,0.54) | 576 (296,859) | 0.23 (0.12,0.34) | 8993 (4515,13360) | 9.14 (4.6,13.56) | 13945 (7177,20904) | 5.34 (2.76,8.01) | -1.41  (-1.9, -0.91) | -1.71  (-2.17 , -1.24) |
| South Asia | 6186 (3330,9063) | 1.06 (0.57,1.56) | 16739 (8631,23927) | 1.12 (0.58,1.61) | 185148 (99645,270388) | 28.61 (15.39,41.85) | 469725 (241739,673435) | 29.62 (15.24,42.45) | 0.47  (-0.26 ,  1.2) | 0.38  (-0.34 , 1.11) |
| Eastern Sub-Saharan Africa | 693 (363,1065) | 0.91 (0.48,1.4) | 1025 (522,1468) | 0.65 (0.33,0.93) | 20325 (10575,31506) | 24.45 (12.77,37.66) | 28947 (14805,41514) | 15.65 (7.97,22.36) | -0.93  (-1.87 , 0.02) | -1.31  (-2.23 , -0.38) |
| Central Sub-Saharan Africa | 86 (39,152) | 0.39 (0.18,0.68) | 252 (101,466) | 0.46 (0.19,0.84) | 2584 (1183,4533) | 10.18 (4.63,17.96) | 7604 (3020,14242) | 11.94 (4.78,22.04) | 0.68  (-0.14 , 1.51) | 0.57  (-0.24 , 1.38) |
| Southern Sub-Saharan Africa | 463 (243,701) | 1.7 (0.9,2.58) | 1068 (543,1556) | 1.86 (0.95,2.68) | 13674 (7151,20680) | 46.05 (24.07,69.57) | 30228 (15317,43759) | 48.06 (24.4,69.84) | 0.4  (-0.34 , 1.15) | 0.28  (-0.5, 1.06) |
| Western Sub-Saharan Africa | 237 (119,352) | 0.28 (0.14,0.42) | 545 (267,815) | 0.3 (0.15,0.45) | 6353 (3194,9376) | 6.85 (3.45,10.14) | 14657 (7088,22043) | 7.01 (3.43,10.46) | 0.53  (-0.22 , 1.28) | 0.38  (-0.33 , 1.1) |
