# Supplementary material for: Triangulating associations between fruit intake and lung cancer risk: evidence from GBD estimates, Mendelian randomization, and real-world validation
Source: Oncologist. 2026 Feb 27;31(7):oyag069. doi: 10.1093/oncolo/oyag069 (PMC13329070; doi:10.1093/oncolo/oyag069)
Supplement: oyag069_Supplementary_Data [file oyag069_supplementary_data.zip › Supplementary Table 4.docx]

| **Supplementary Table 4 Baseline Characteristics of Participants** | | | | |
| --- | --- | --- | --- | --- |
| **Patient Characteristics** | **N=641**  **(%)** | **Lung malignancies**  **N= 295 (%)** | **Benign lung nodule**  **N= 346 (%)** | **P -value** |
| **Age** |  |  |  | ＜0.0001 |
| ≤65 | 357(56%) | 133(45%) | 224(65%) |  |
| >65 | 284(44%) | 162(55%) | 122(35%) |  |
| **Sex** |  |  |  | 0.3616 |
| Male | 226(35%) | 110(37%) | 116(34%) |  |
| Female | 415(65%) | 185(63%) | 230 (66%) |  |
| **Fruit intake** |  |  |  | 0.0079 |
| Q1 | 161(25%) | 89(30%) | 72(21%) |  |
| Q2 | 163(25%) | 80(27%) | 83(24%) |  |
| Q3 | 157(25%) | 66(23%) | 91(26%) |  |
| Q4 | 160(25%) | 60(20%) | 100(29%) |  |
| **Smoking** |  |  |  | 0.0031 |
| Yes | 209(33%) | 114(39%) | 95(27%) |  |
| No | 432(67%) | 181(61%) | 251(73%) |  |
| **Drinking** |  |  |  | 0.1705 |
| Yes | 160(25%) | 66(22%) | 94(27%) |  |
| No | 481(75%) | 229(78%) | 252(73%) |  |
| **BMI** |  |  |  | 0.9413 |
| <18.5 | 20(3%) | 9(3%) | 11(3%) |  |
| 18.5≤BMI<24.0 | 323(50%) | 149(51%) | 174(51%) |  |
| 24.0≤BMI<28.0 | 230(36%) | 108(37%) | 122(35%) |  |
| ≥28.0 | 68(11%) | 29(9%) | 39(11%) |  |
| **Marriage** |  |  |  | 0.0050 |
| Unmarried | 23(4%) | 3(1%) | 20(6%) |  |
| Married | 566(88%) | 266(90%) | 300(87%) |  |
| Divorced | 18(3%) | 7(2%) | 11(3%) |  |
| Widowed | 34(5%) | 19(7%) | 15(4%) |  |
| **Education** |  |  |  | ＜0.0001 |
| Junior high school and below | 339(53%) | 183(62%) | 156(45%) |  |
| High school and technical secondary school | 145(23%) | 68(23%) | 77(22%) |  |
| junior college | 87(14%) | 30(10.3%) | 57(16%) |  |
| undergraduate | 60(9%) | 13(4.4%) | 47(14%) |  |
| graduate and above | 10(1%) | 1(0.3%) | 9(3%) |  |
| **Residence** |  |  |  | 0.0490 |
| Urban | 441(69%) | 191(65%) | 250(72%) |  |
| Rural | 200(31%) | 104(35%) | 96(28%) |  |
| **Annual per capita income** | |  |  | 0.0008 |
| <36000 | 336(52%) | 173(59%) | 163(47%) |  |
| 36000-60000 | 229(36%) | 102(35%) | 127(37%) |  |
| 60000-120000 | 61(10%) | 17(5%) | 44(13%) |  |
| ≥120000 | 15(2%) | 3(1%) | 12(3%) |  |
| **Difficulty falling asleep** | |  |  | 0.1088 |
| Yes | 271(42%) | 135(46%) | 136(39%) |  |
| No | 370(58%) | 160(54%) | 210(61%) |  |
| Fruit intake: Q1: <25%; Q2:25%-50%; Q3:50%-75%; Q4:>75% | | | | |
| BMI: BMI<18.5: wizened; 18.5≤BMI<24.0: normal; 24.0≤BMI<28.0: overweight; BMI≥28: obesity | | | | |
