# Supplementary material for: Triangulating associations between fruit intake and lung cancer risk: evidence from GBD estimates, Mendelian randomization, and real-world validation
Source: Oncologist. 2026 Feb 27;31(7):oyag069. doi: 10.1093/oncolo/oyag069 (PMC13329070; doi:10.1093/oncolo/oyag069)
Supplement: oyag069_Supplementary_Data [file oyag069_supplementary_data.zip › Supplementary Table 5.docx]

| **Supplementary Table 5 Univariate Analysis of Confounding Factors on Fruit Intake** | | | | |
| --- | --- | --- | --- | --- |
| **Variable** | **Levels** | **chi2** | **Dof** | **p_value** |
| Income | 4 | 36.29126502 | 9 | ＜0.001 |
| Smoking | 2 | 22.23908822 | 3 | ＜0.001 |
| Education | 5 | 30.3996575 | 12 | 0.002 |
| Residential location | 2 | 10.81724837 | 3 | 0.013 |
| Gender | 2 | 10.09457401 | 3 | 0.018 |
| Drinking | 2 | 5.710159852 | 3 | 0.127 |
| BMI | 4 | 13.87763661 | 9 | 0.127 |
| Age | 2 | 3.417893674 | 3 | 0.332 |
| Marriage | 4 | 8.946524396 | 9 | 0.442 |
| Sleep quality | 2 | 1.249489832 | 3 | 0.741 |
