# Supplementary material for: Triangulating associations between fruit intake and lung cancer risk: evidence from GBD estimates, Mendelian randomization, and real-world validation
Source: Oncologist. 2026 Feb 27;31(7):oyag069. doi: 10.1093/oncolo/oyag069 (PMC13329070; doi:10.1093/oncolo/oyag069)
Supplement: oyag069_Supplementary_Data [file oyag069_supplementary_data.zip › Supplementary Table 6.docx]

| **Supplementary Table 6 Spearman's analysis of the Influence of Confounding Factors on Fruit Intake** | | |
| --- | --- | --- |
| **variable** | **spearman** | **p_value** |
| Income | 0.223842 | ＜0.001 |
| Education | 0.188514 | ＜0.001 |
| Smoking | -0.18442 | ＜0.001 |
| Residential location | -0.12239 | 0.002 |
| Gender | -0.11809 | 0.003 |
| Age | -0.073 | 0.065 |
| Drinking | -0.0545 | 0.168 |
| Sleep quality | -0.04116 | 0.298 |
| Marriage | -0.0357 | 0.367 |
