# Supplementary material for: Triangulating associations between fruit intake and lung cancer risk: evidence from GBD estimates, Mendelian randomization, and real-world validation
Source: Oncologist. 2026 Feb 27;31(7):oyag069. doi: 10.1093/oncolo/oyag069 (PMC13329070; doi:10.1093/oncolo/oyag069)
Supplement: oyag069_Supplementary_Data [file oyag069_supplementary_data.zip › Supplementary Table 7.docx]

| **Supplementary Table 7 Multivariate Ordered Logistic Regression Analysis of Confounding Factors' Effects on Fruit Intake** | | | | |
| --- | --- | --- | --- | --- |
| **Term** | **OR** | **CI_lower** | **CI_upper** | **p_value** |
| Age | 1.06 | 0.79 | 1.44 | 0.694 |
| Gender | 0.80 | 0.53 | 1.20 | 0.286 |
| Marriage | 1.05 | 0.79 | 1.38 | 0.743 |
| Education | 1.13 | 0.96 | 1.33 | 0.149 |
| BMI | 0.94 | 0.78 | 1.14 | 0.516 |
| Residential location | 0.89 | 0.64 | 1.24 | 0.483 |
| Income | 1.50 | 1.20 | 1.86 | ＜0.001 |
| Sleep quality | 0.81 | 0.60 | 1.09 | 0.158 |
| Smoking | 0.63 | 0.42 | 0.94 | 0.022 |
