# Supplementary figures and images for: Triangulating associations between fruit intake and lung cancer risk: evidence from GBD estimates, Mendelian randomization, and real-world validation
Source: Oncologist. 2026 Feb 27;31(7):oyag069. doi: 10.1093/oncolo/oyag069 (PMC13329070; doi:10.1093/oncolo/oyag069)

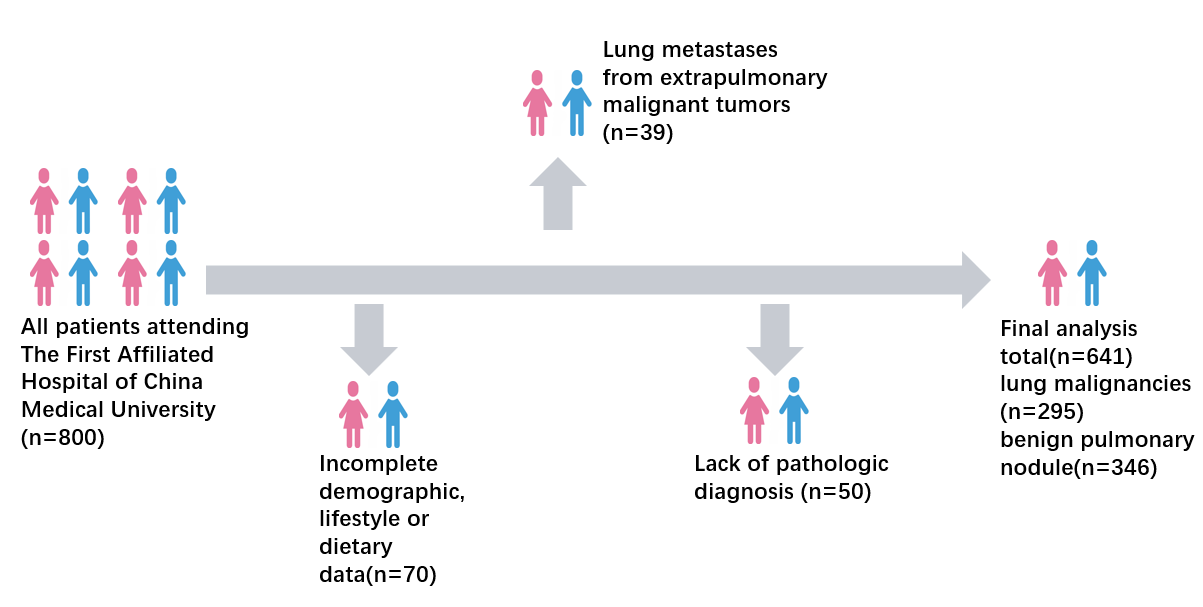

Supplement: oyag069_Supplementary_Data [file oyag069_supplementary_data.zip › Supplementary Figure1.png]

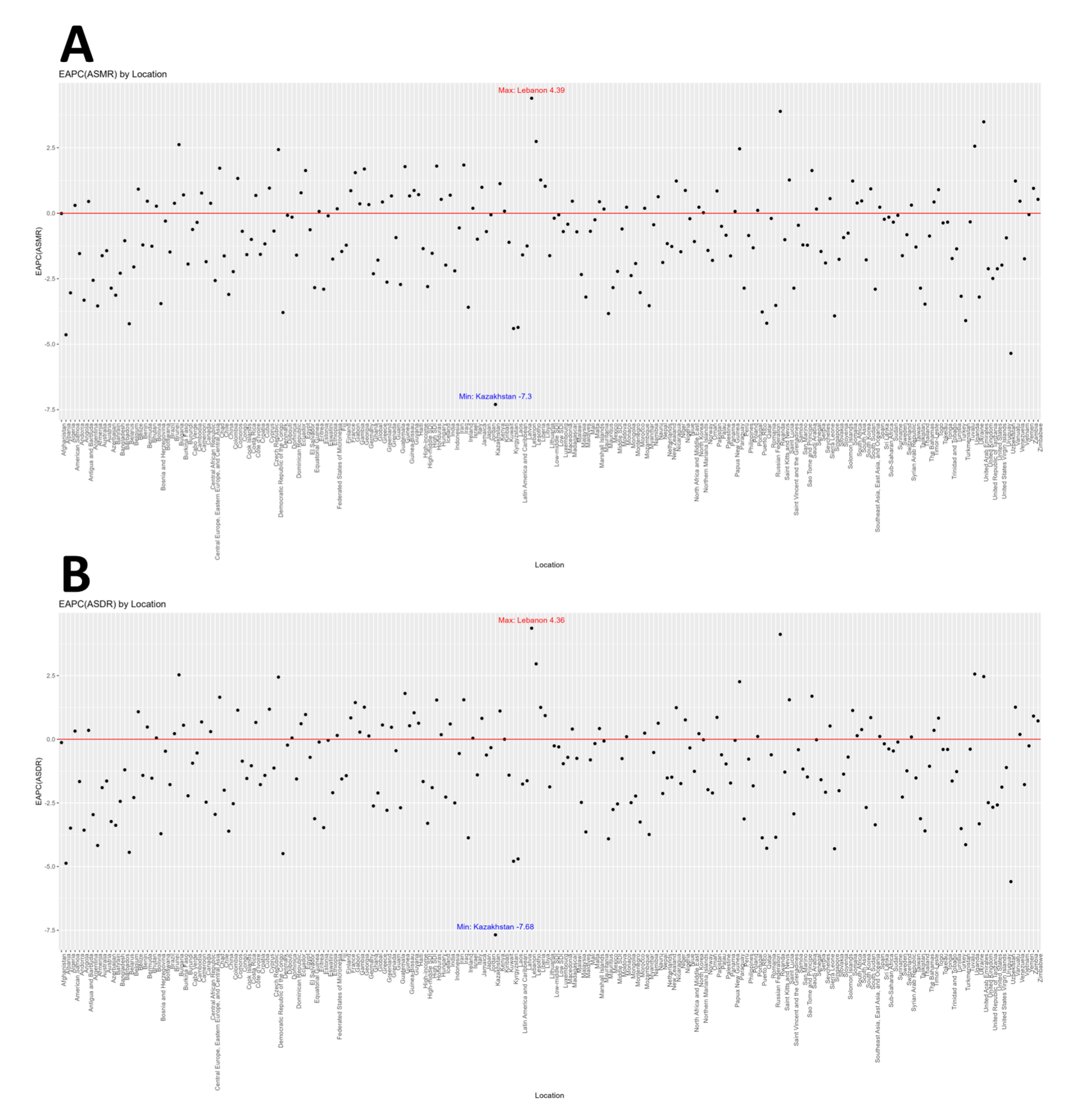

Supplement: oyag069_Supplementary_Data [file oyag069_supplementary_data.zip › Supplementary Figure2.png]

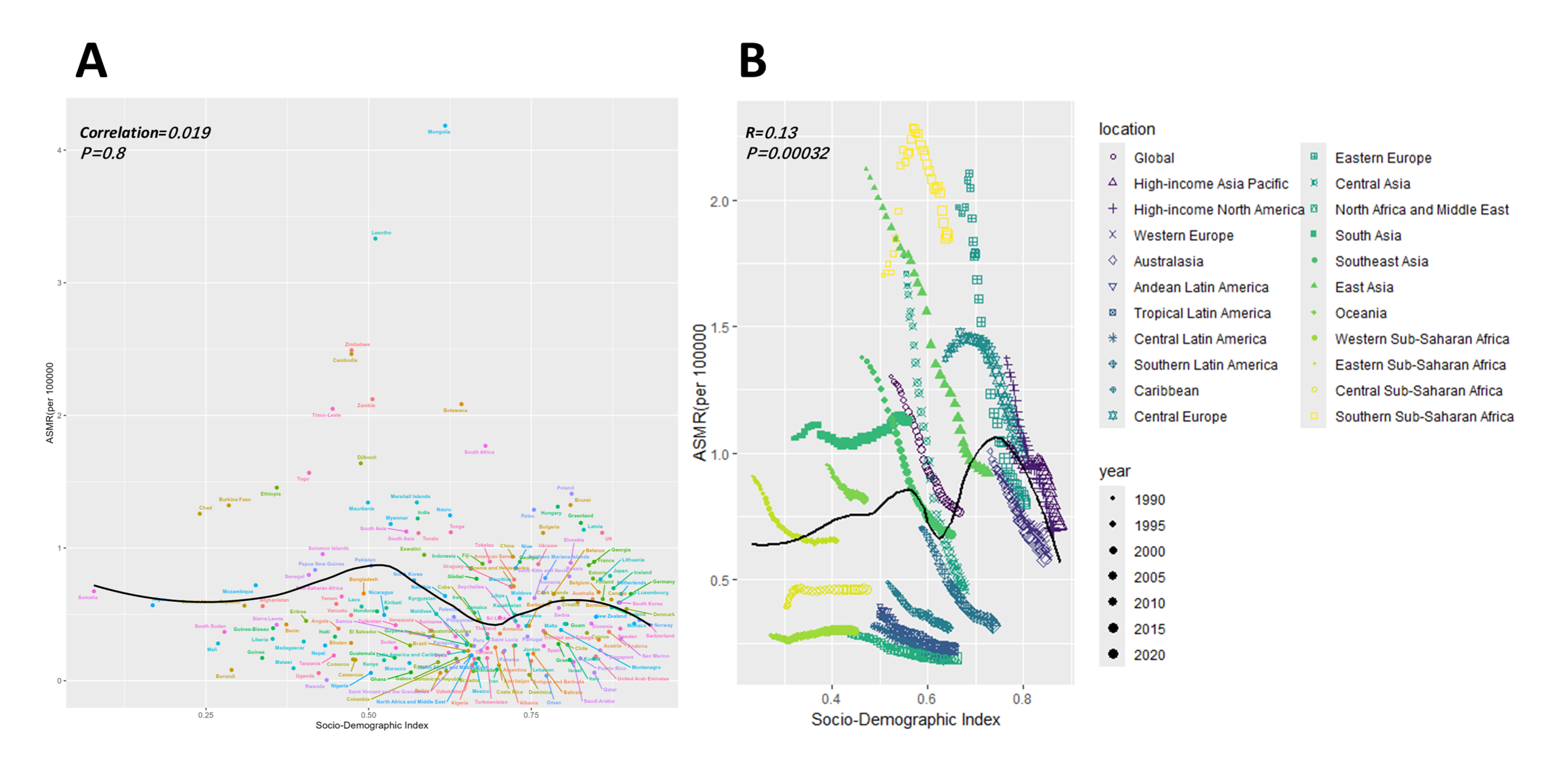

Supplement: oyag069_Supplementary_Data [file oyag069_supplementary_data.zip › Supplementary Figure3.png]

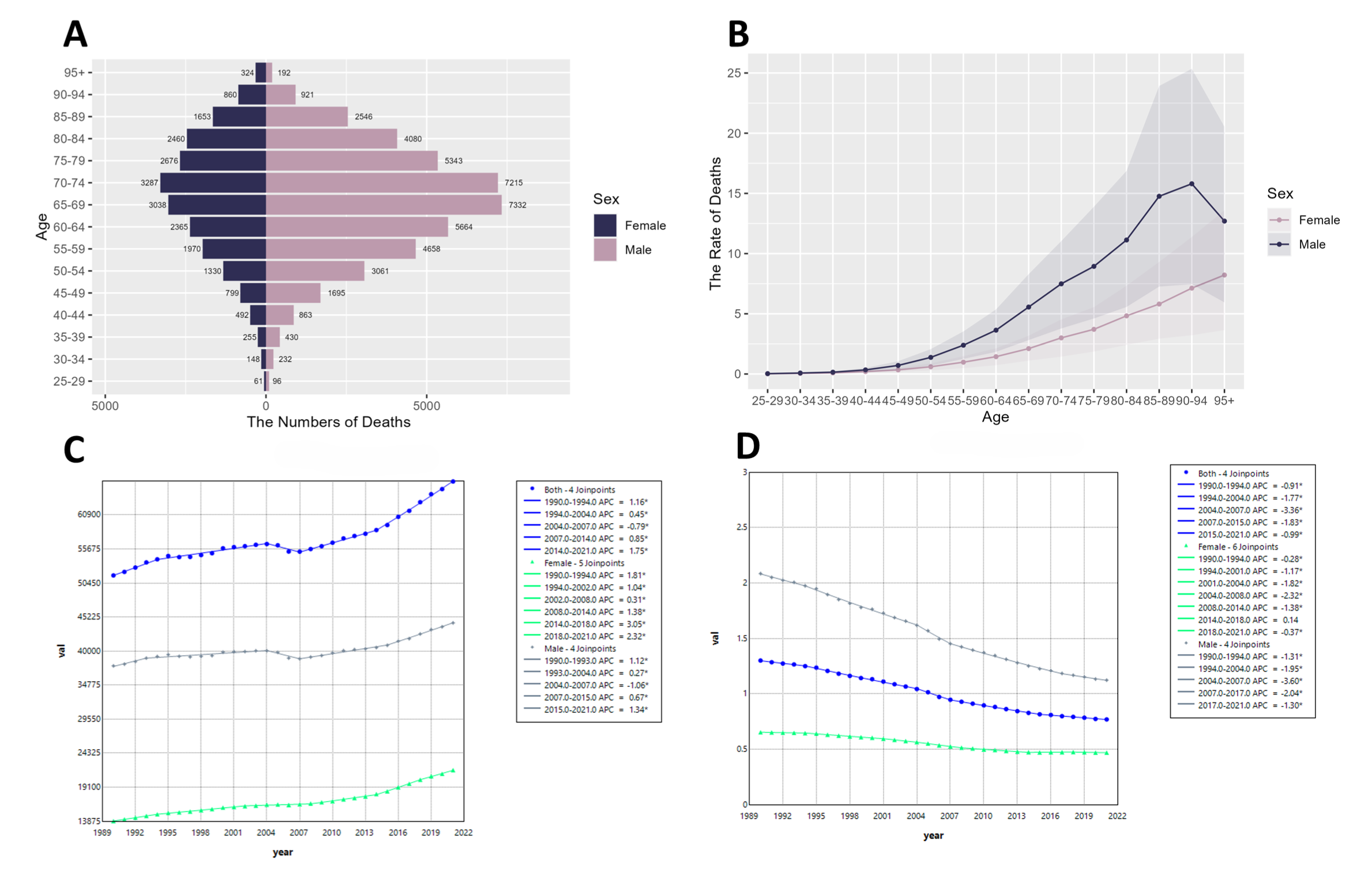

Supplement: oyag069_Supplementary_Data [file oyag069_supplementary_data.zip › Supplementary Figure4.png]

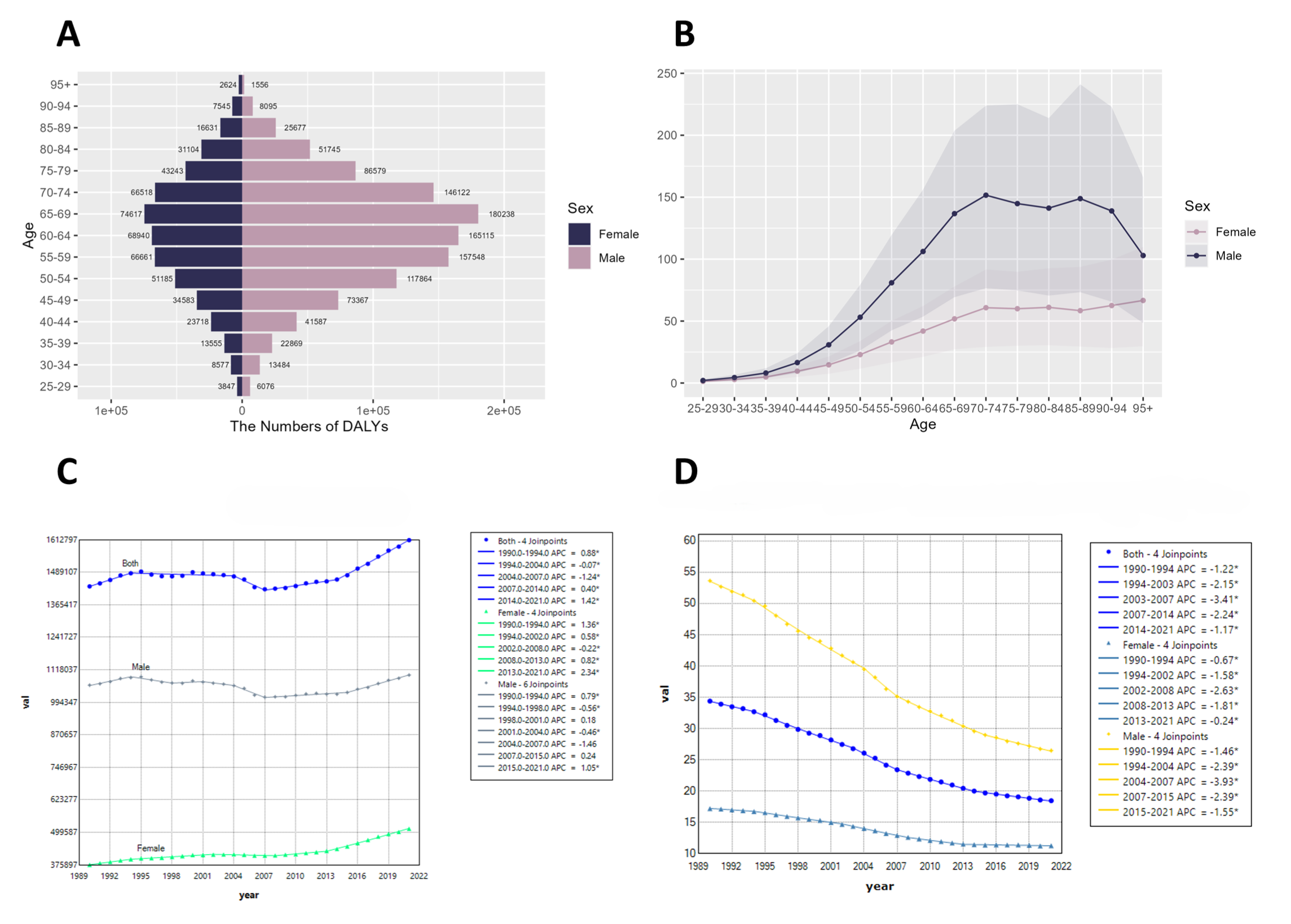

Supplement: oyag069_Supplementary_Data [file oyag069_supplementary_data.zip › Supplementary Figure5.png]

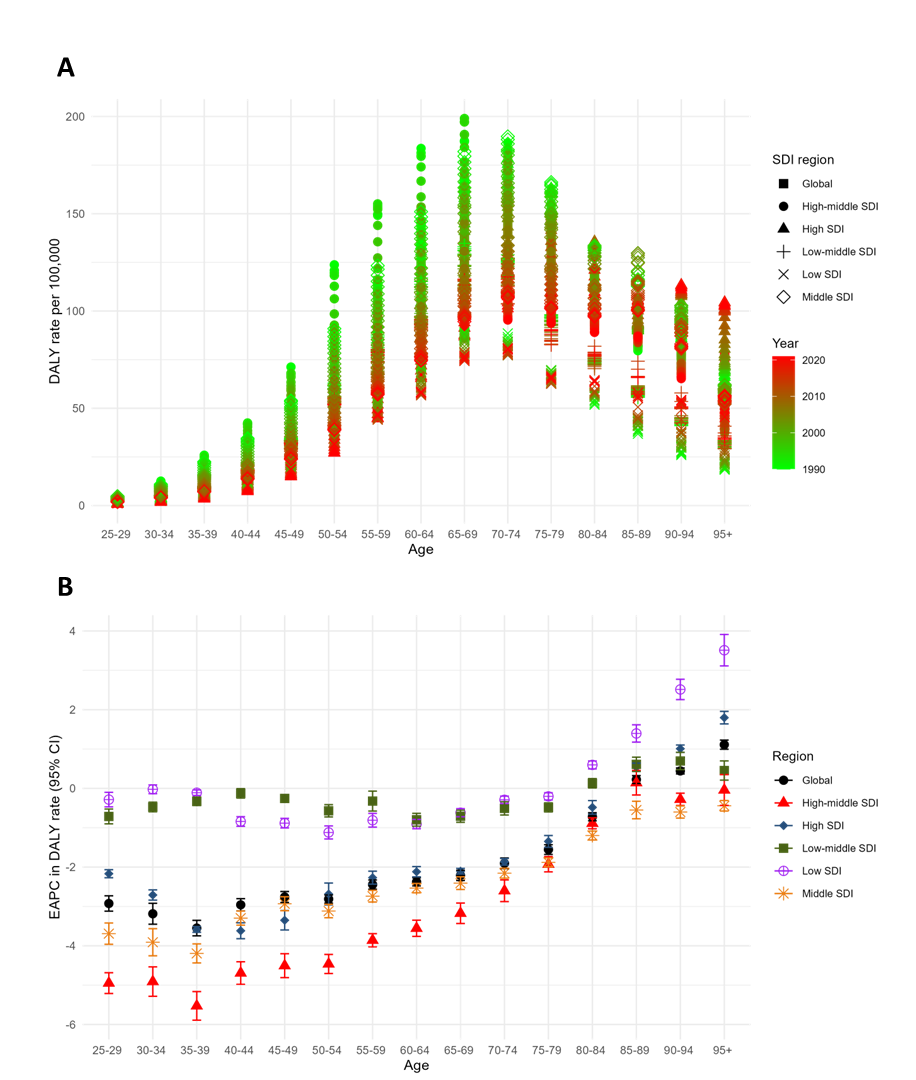

Supplement: oyag069_Supplementary_Data [file oyag069_supplementary_data.zip › Supplementary Figure6.png]

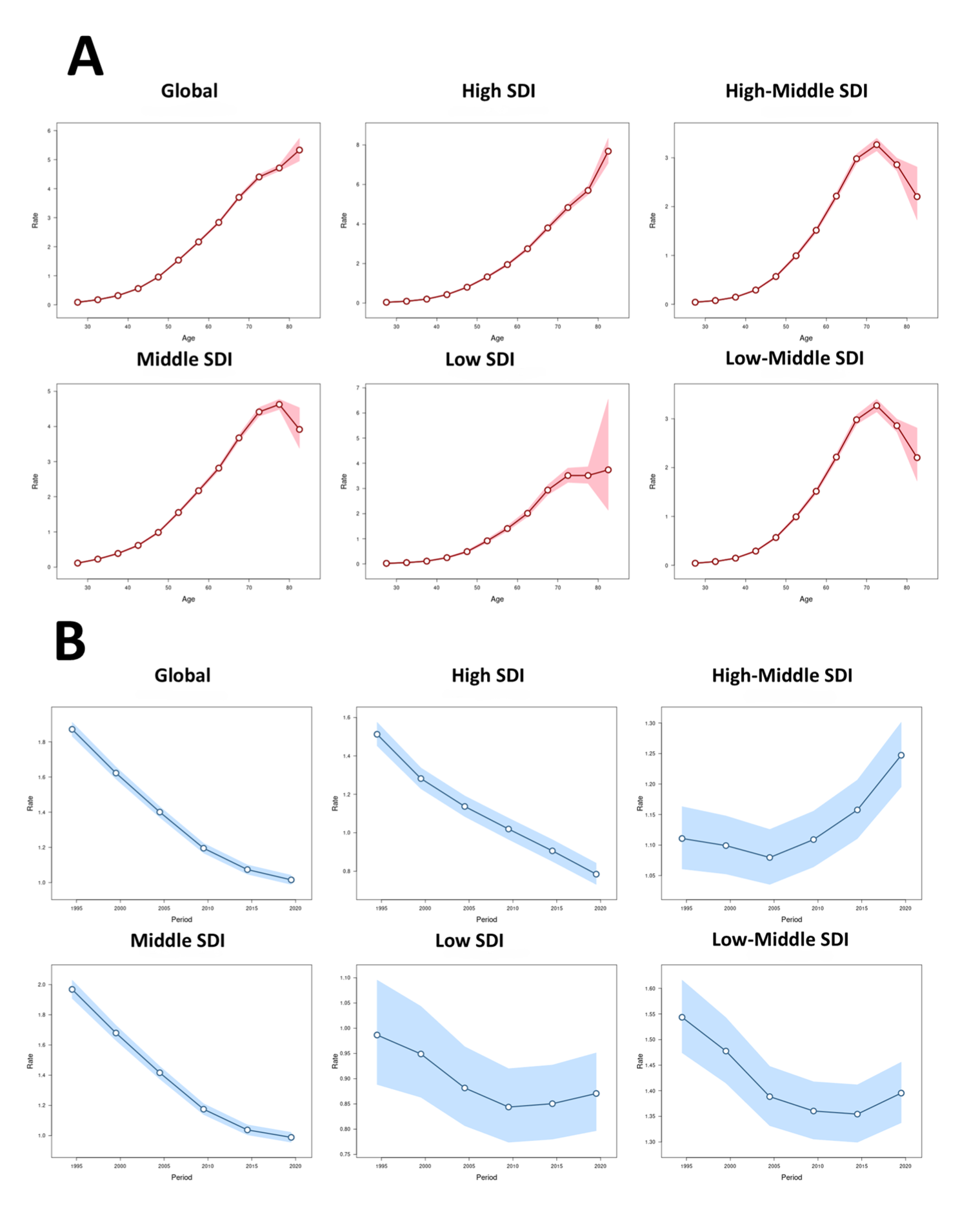

Supplement: oyag069_Supplementary_Data [file oyag069_supplementary_data.zip › Supplementary Figure7.png]

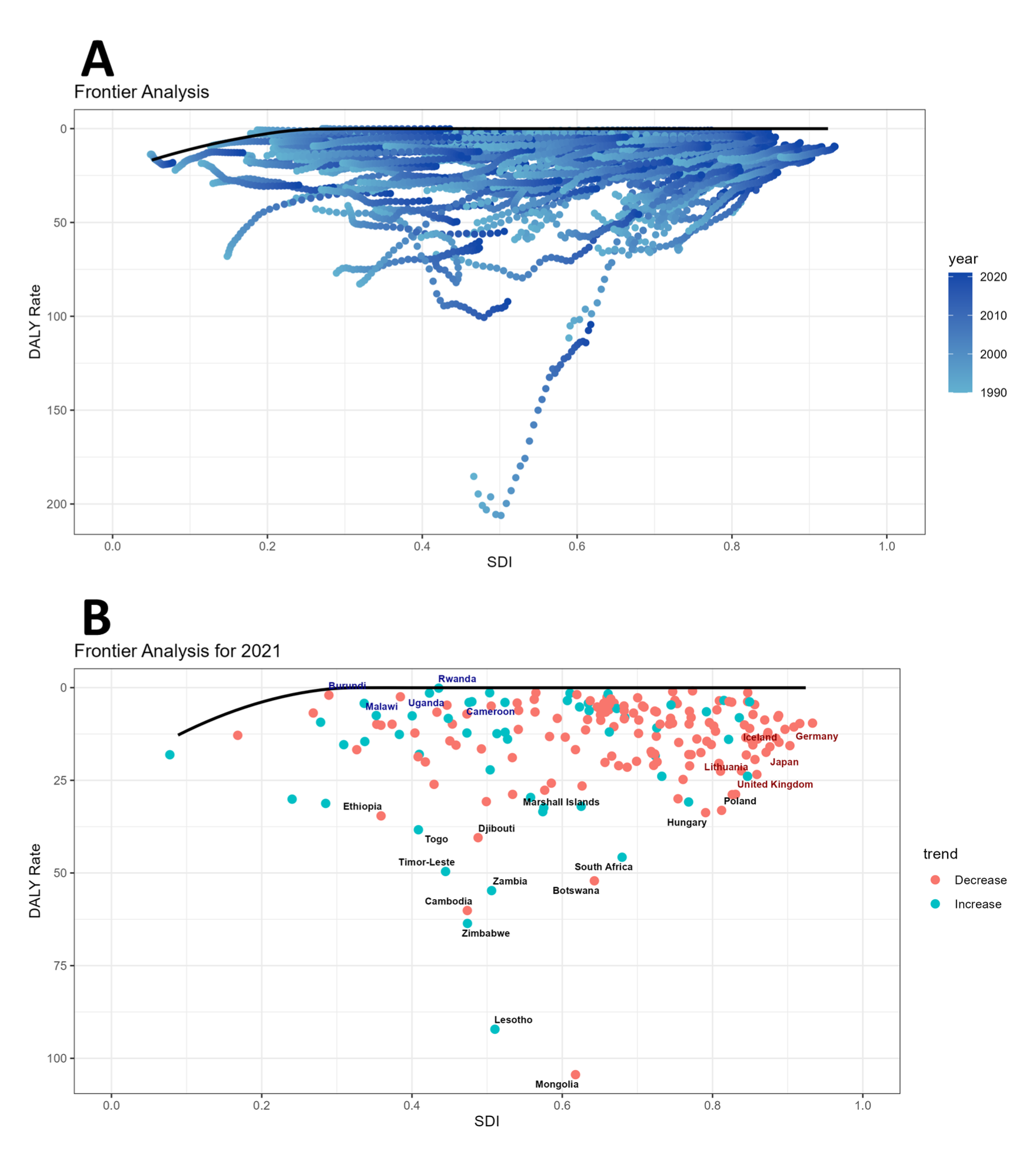

Supplement: oyag069_Supplementary_Data [file oyag069_supplementary_data.zip › Supplementary Figure8.png]

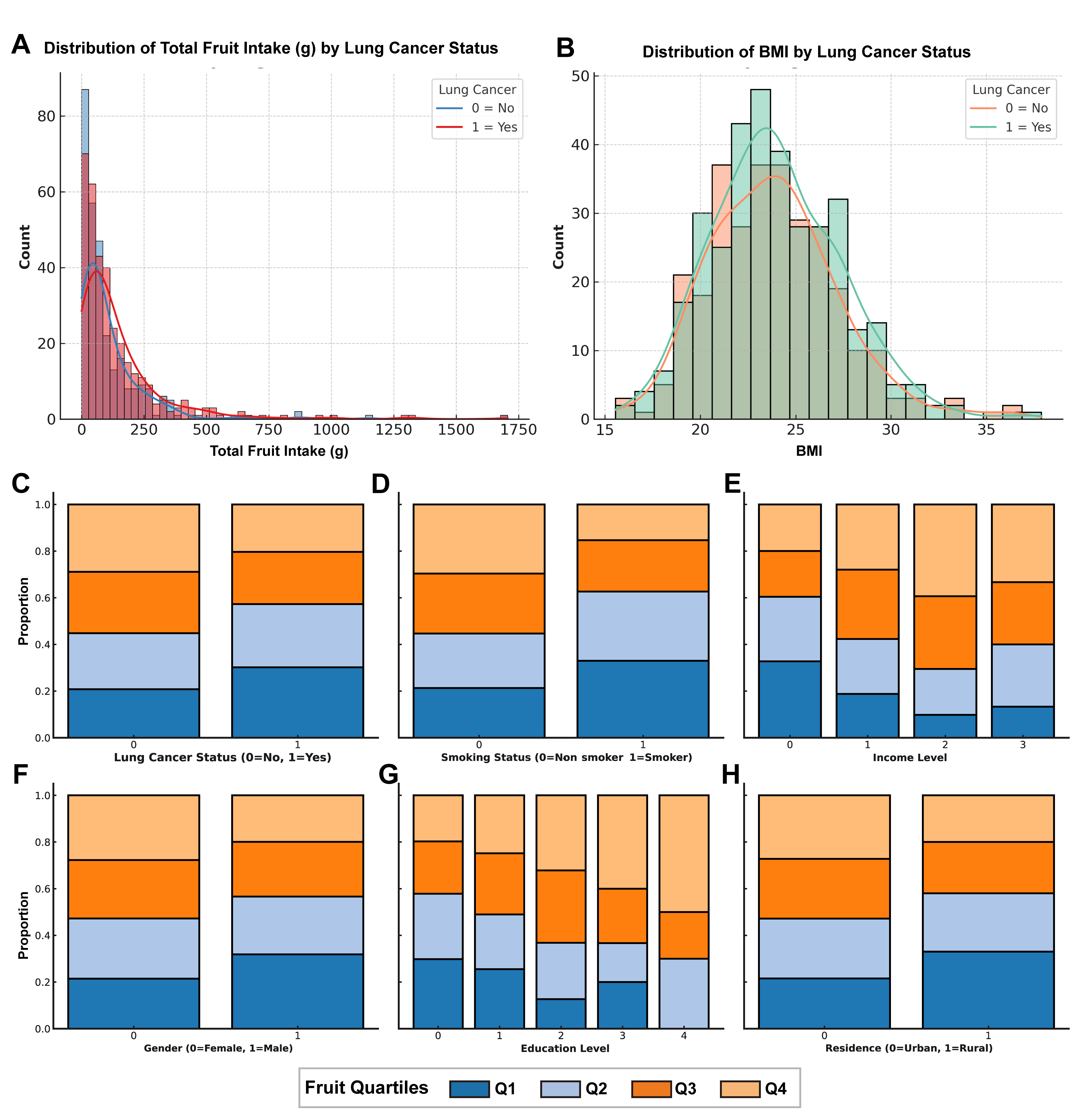

Supplement: oyag069_Supplementary_Data [file oyag069_supplementary_data.zip › Supplementary Figure9.tif]

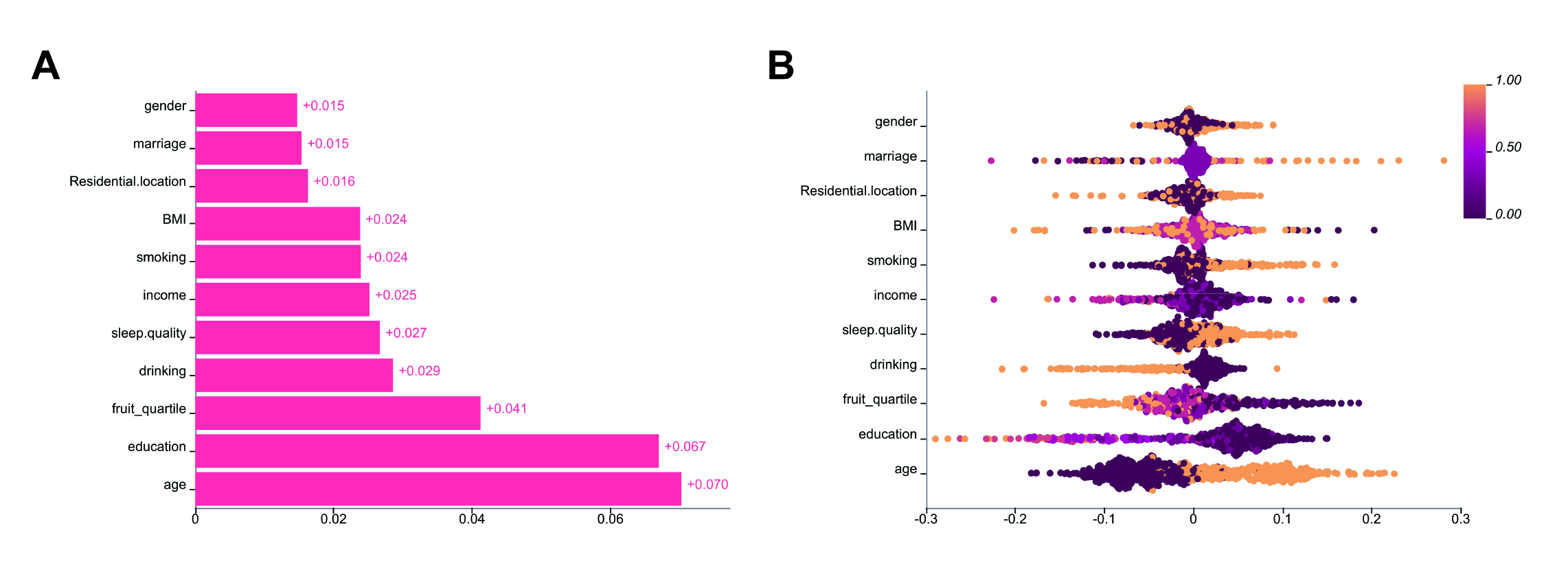

Supplement: oyag069_Supplementary_Data [file oyag069_supplementary_data.zip › Supplementary Figure10.tif]

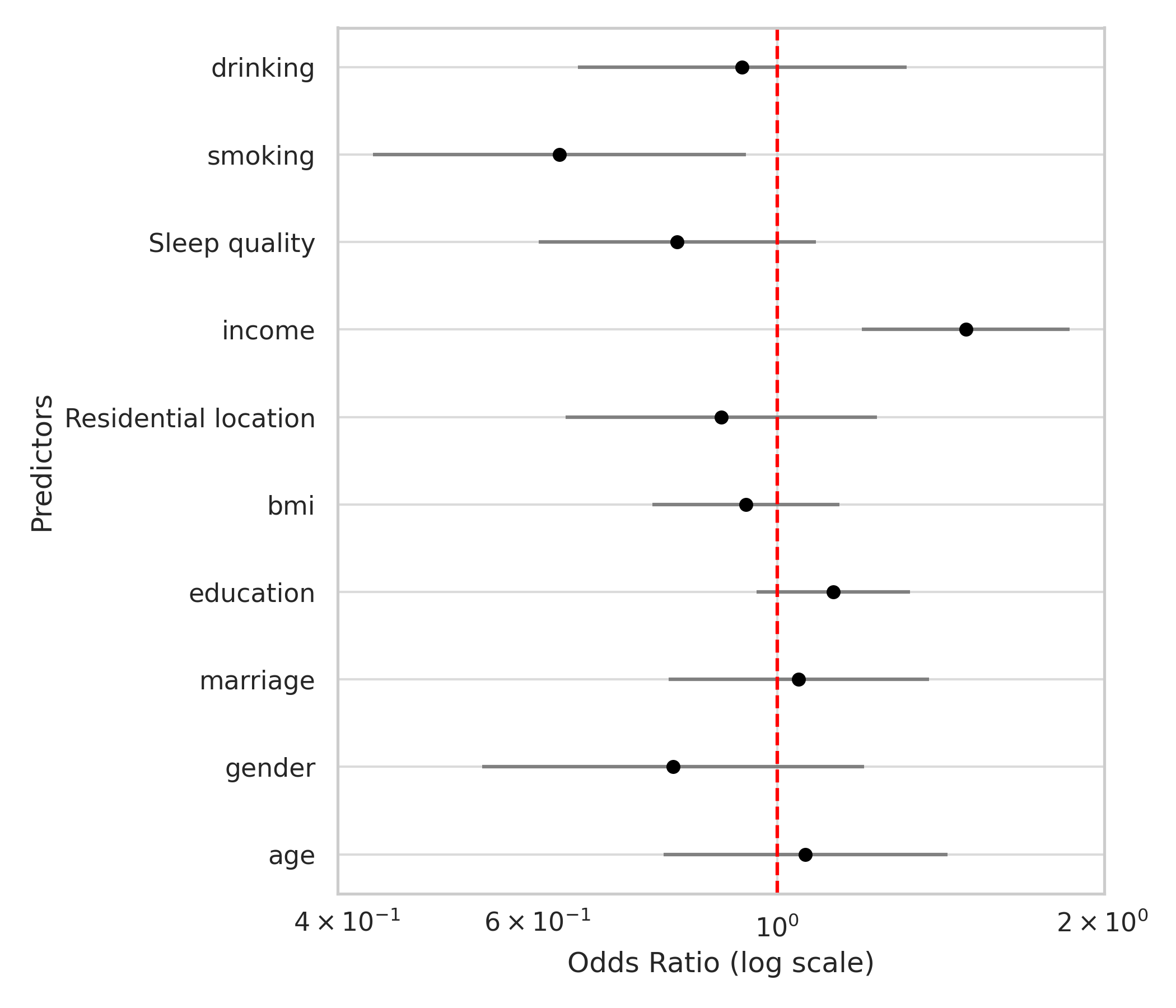

Supplement: oyag069_Supplementary_Data [file oyag069_supplementary_data.zip › Supplementary Figure11.png]

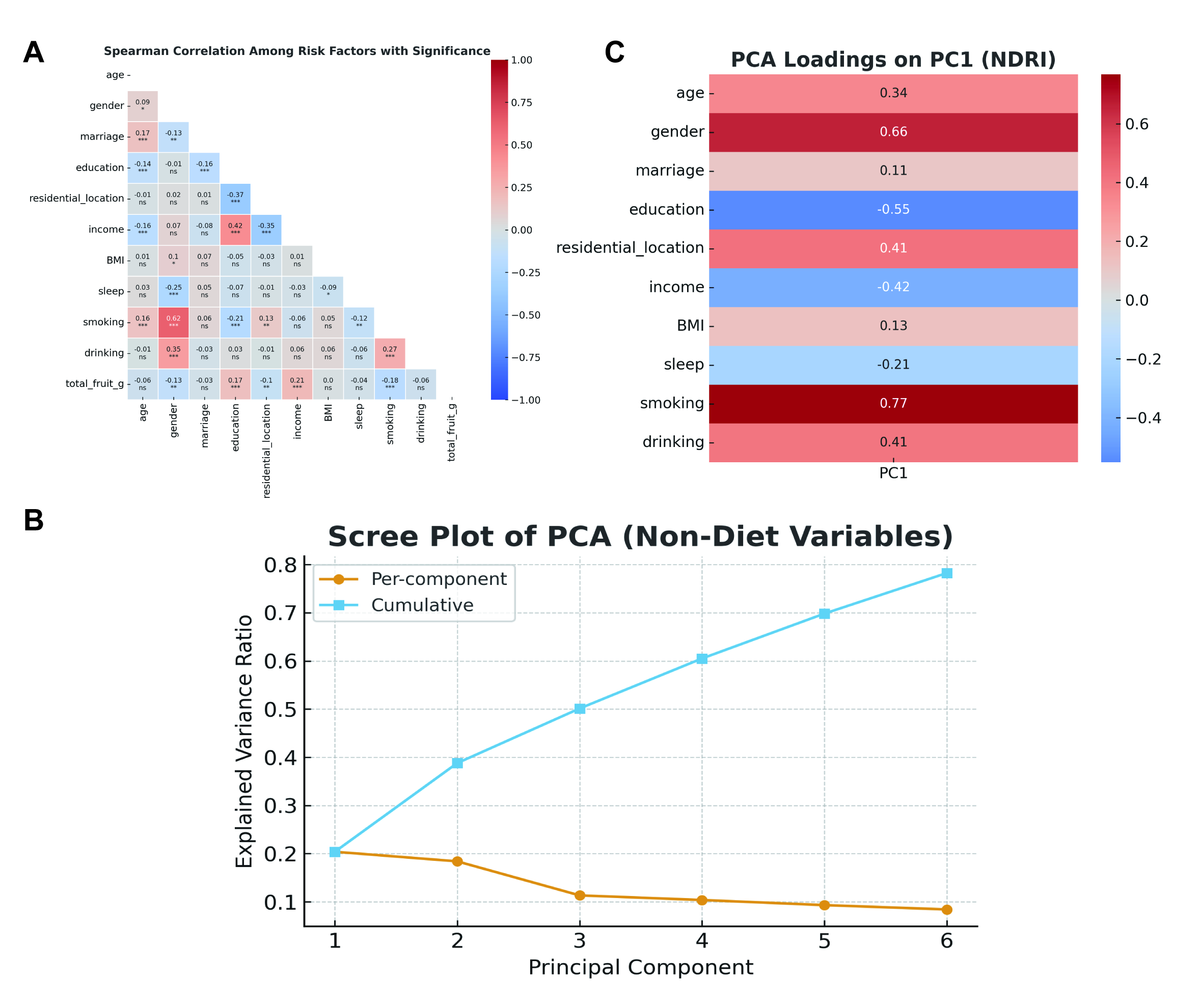

Supplement: oyag069_Supplementary_Data [file oyag069_supplementary_data.zip › Supplementary Figure12.tif]

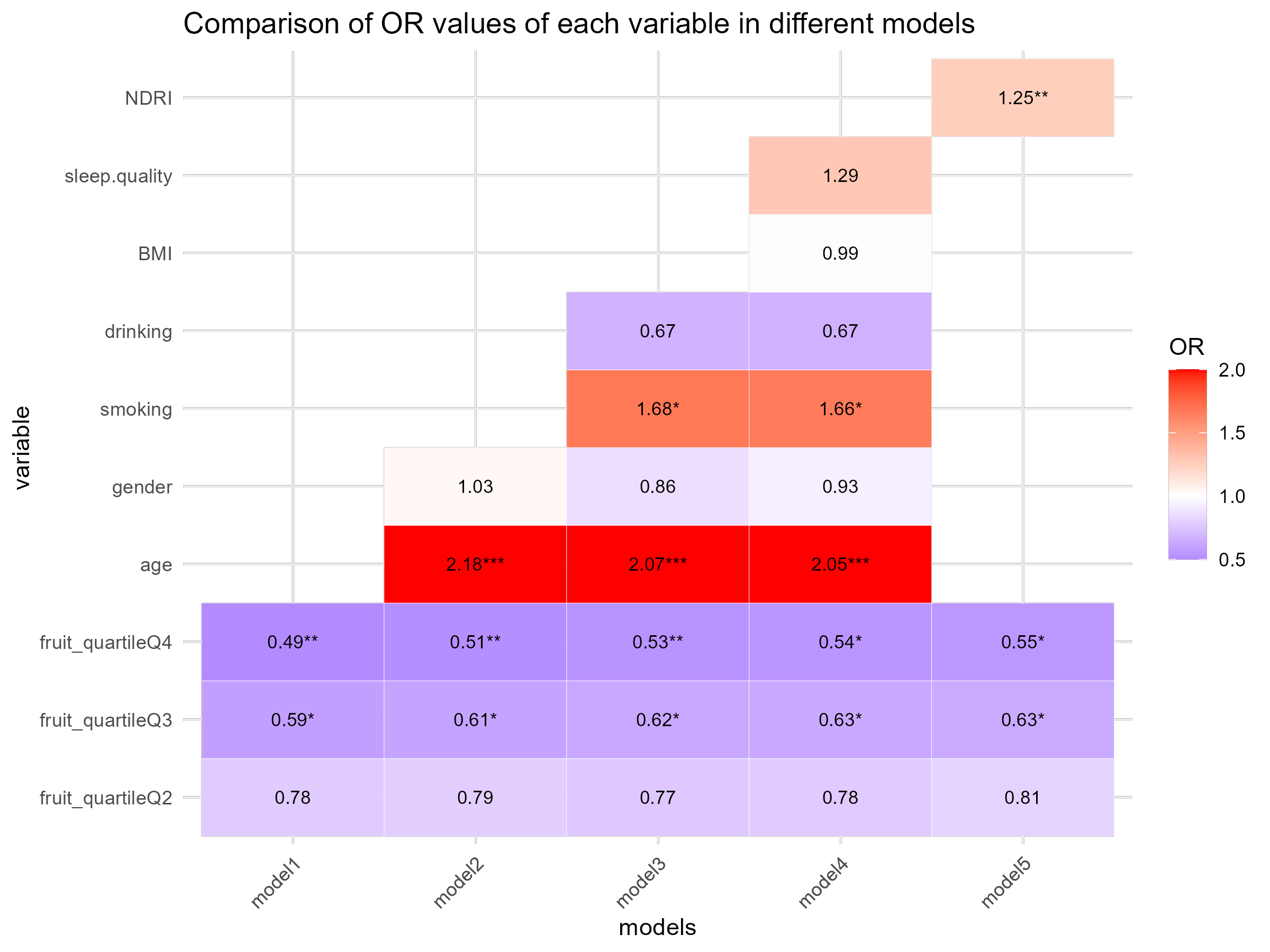

Supplement: oyag069_Supplementary_Data [file oyag069_supplementary_data.zip › Supplementary Figure13.tiff]

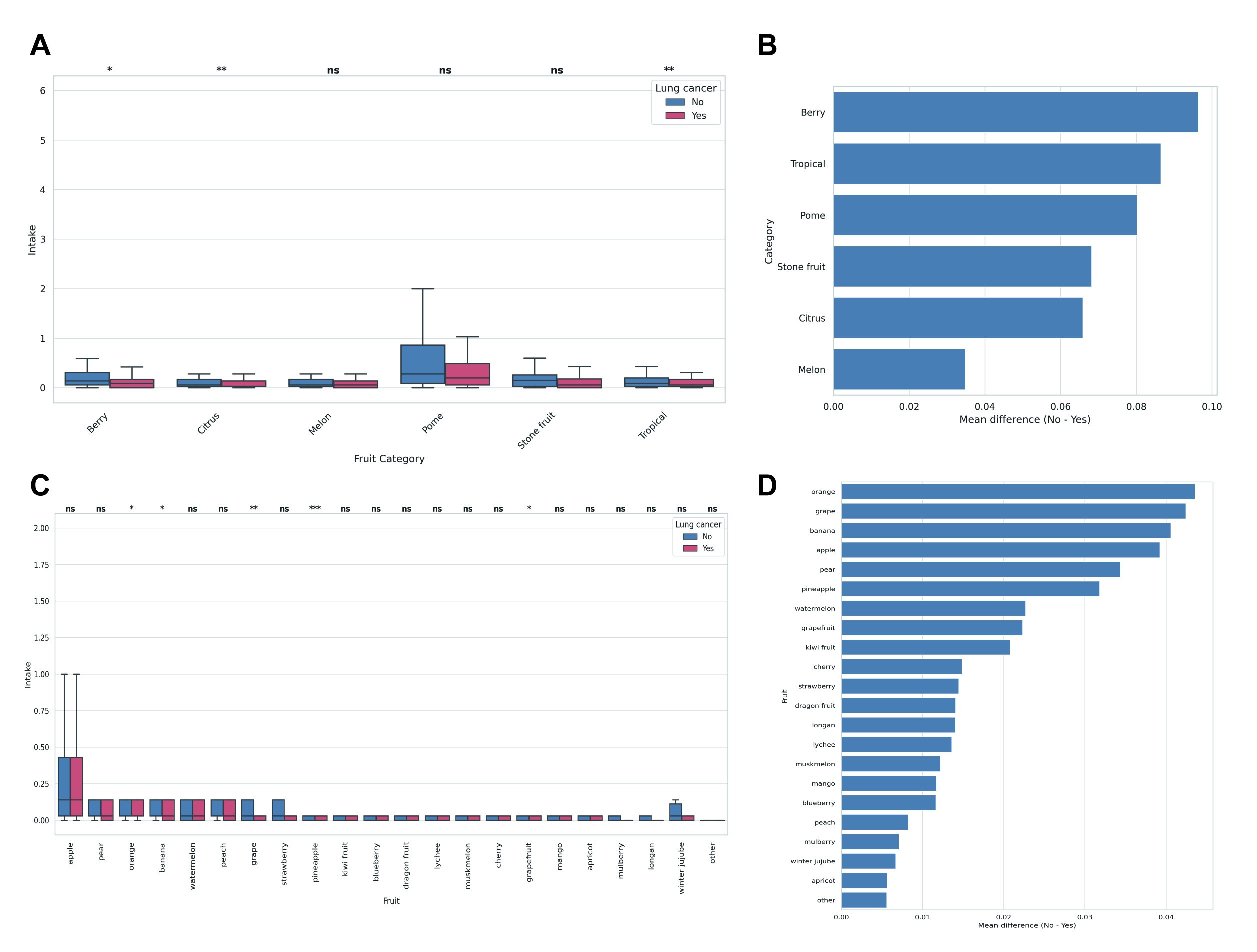

Supplement: oyag069_Supplementary_Data [file oyag069_supplementary_data.zip › Supplementary Figure14.tif]
